# Wearable Technology, Smart Home Systems, and Mobile Apps for the Self‑Management of Patient Outcomes in Dementia Care: Systematic Review

**DOI:** 10.2196/65385

**Published:** 2025-08-21

**Authors:** Gary Cornelius, William Hodgson, Roma Maguire, Kieren Egan

**Affiliations:** 1 Department of Computer & Information Sciences (CIS) Digital Health and Wellness Research Group University of Strathclyde Glasgow United Kingdom; 2 Department of Physical Activity for Health University of Strathclyde Glasgow United Kingdom

**Keywords:** Alzheimer disease, vascular dementia, frontotemporal dementia, Lewy body, smartwatch, wearable, smart home, mobile apps, quality of life, behavior control, personalized care and self-management

## Abstract

**Background:**

The dementia landscape has evolved, with earlier diagnoses, improved prevention understanding (eg, modifiable factors), and new treatments. Emerging digital technologies (eg, wearables, smart home systems, and mobile apps) offer self‑management opportunities; yet, gaps persist regarding integration into the care needs and preferences of people with dementia. Broader gaps remain concerning intervention design; adaptation; and implementation, including effectiveness, study quality, and accessibility.

**Objective:**

This systematic review aims to synthesize and critically appraise existing literature on digital self-management technologies (wearables, smart home systems, and mobile apps) intended to reduce dementia-associated behaviors, enhance self-management, and improve quality of life (QoL). It evaluates intervention characteristics, effectiveness, accessibility, study design, and methodological quality according to international standards.

**Methods:**

A systematic search across 9 databases (PubMed, Scopus, ACM Digital Library, CINAHL, PsycInfo, Web of Science, IEEE Xplore, Embase, and MEDLINE) identified relevant English‑language studies published between January 1, 2013, and September 30, 2023. Search terms covered dementia, QoL, behavioral and self‑management strategies, and digital technologies. Eligible studies involved adults with dementia using wearable, smart home, or mobile technologies targeting QoL, behavior, and autonomy. Two reviewers independently appraised study design, hardware, and intervention purpose. Outcomes were mapped to the Nursing Outcomes Classification and benchmarked against National Institute for Health and Care Excellence quality standard 184. Accessibility was evaluated by availability, cost, usability, and context. Bias mitigation included a PRISMA (Preferred Reporting Items for Systematic Reviews and Meta-Analyses)‑guided strategy and PROSPERO registration. Methodological quality and bias were assessed using the Mixed Methods Appraisal Tool, the Critical Appraisal Skills Program, and a bespoke characterization framework.

**Results:**

Twenty-four studies evaluated interventions based on wearables, smart home systems, or apps for people with dementia and carers. Outcomes centered on neurocognition (24/24, 100%), self-care (17/24, 71%), and health behavior (13/24, 54%). Identified needs included managing distress (15/24, 62%) and supporting carers (15/24, 62%). Technologies included commercial tools (activity trackers, health-based wearables, and digital prompters) but were often inaccessible due to complex setup requirements and ongoing support needs. Substantial methodological heterogeneity precluded meta-analysis, necessitating narrative synthesis. Study quality was generally good to excellent, but samples were small, reporting incomplete, and outcomes unblinded. Only 1 (17%) of 6 randomized controlled trials reported effect sizes, indicating moderate decline in QoL at 24 months; effectiveness in other studies remains uncertain.

**Conclusions:**

Research on digital technologies for dementia self‑management shows benefits, particularly with off‑the‑shelf devices and mobile apps supporting person‑centered outcomes. Notable limitations include inadequate participant diversity (eg, atypical dementias and minoritized populations) and insufficient high‑quality research on QoL and behavioral outcomes, such as symptom management and self‑control. Future research must prioritize innovative solutions enhancing accessibility and usability, emphasizing simplified configuration, personalized adaptability, and effective training and support structures.

**Trial Registration:**

PROSPERO CRD42023461841; https://www.crd.york.ac.uk/PROSPERO/view/CRD42023461841

## Introduction

### Background

Currently, >55 million people worldwide have dementia, costing global economies US $1.3 trillion, and this cost is expected to rise to US $2.8 trillion by 2030 [1]. As populations age, the number of people with dementia is expected to triple by 2050, leading to increased demand for resources to support people with dementia at a time when the health care workforce is projected to shrink due to retirement and aging. Consequently, there is a growing need for technological solutions.

Dementias are chronic and irreversible brain disorders that lead to severe cognitive impairments and ultimately death. These disorders disrupt cognitive functions such as memory, decision-making, and personality, significantly diminishing patients’ quality of life (QoL). The early signs include memory loss, challenges in planning or problem-solving, difficulty completing familiar tasks, confusion with time or place, trouble understanding visual images, problems with words, misplacing things, decreased judgment, withdrawal from work or social activities, and changes in mood or personality [2]. Alzheimer disease, a form of dementia, is frequently perceived as one of the most feared conditions in later life. This perception stems from its association with cognitive decline, loss of autonomy, and the prolonged emotional and financial burden it imposes on families. A 2012 YouGov poll commissioned by Alzheimer’s Society and Saga Homecare revealed that 63% of British adults expressed concern about dementia, with the highest levels of worry among those aged ≥55 years (66%). Notably, even among younger adults aged 18 to 24 years, 61% reported being worried about the condition [3].

Dementias are complex, progressive conditions characterized by diverse and evolving needs, which differ significantly from person to person and change over the course of the illness. With dementia diagnoses occurring increasingly earlier, individuals now have greater capacity to actively participate in managing their condition. Concurrently, rapid advancements in digital technologies, such as wearables, smart home systems, and mobile apps, have introduced numerous self-management possibilities. Digital self-management interventions are designed to be used by people with dementia themselves, supporting their daily functioning, autonomy, and well-being. While these technologies may initially focus on reducing risk and promoting independence, they can also evolve to assist with memory recall, decision-making, communication, and meaningful engagement. However, the vast array of available technologies can be overwhelming for individuals living with dementia. Moreover, most existing research predominantly addresses technology-based interventions aimed at health care professionals or caregivers, rather than directly supporting individuals with dementia themselves.

Therefore, there is a critical need for a systematic exploration of technology options specifically tailored to support self-management by people with dementia. This includes identifying interventions that are effective, accessible, and suitably adapted to the evolving capabilities and preferences of individuals as their dementia progresses, while also respecting issues of privacy, data security, and informed consent. Addressing these gaps will ensure that technology interventions are appropriately integrated into personalized care pathways, thereby enhancing QoL and dementia‑associated behaviors, empowering individuals with dementia to better manage their condition.

Wearable devices [4], mobile apps [5,6], and smart home technologies [7] have emerged as useful tools for monitoring dementia‑associated behavior, encouraging behavioral change [8], and enhancing QoL for people living with dementia [9]. These technologies are accessible, scalable, and capable of supporting large populations [10-12]. In addition, they are personal devices that can be individually customized, making them particularly useful for applications in telehealth, mobile health, and personalized medicine. These technologies have various applications in health, such as continuously monitoring vital signs; assisting with medication compliance, fall detection, and hearing loss detection; and providing timely warnings or reminders.

Further innovations, such as blood-based markers, biosensing, smart home monitoring, and electronic records, are leading to significant advances in assistive technologies that support personal behavior change. These advances are crucial because patient-modifiable risk factors are understood to be responsible for 40% of cases of dementia and to modify disease progression [13,14]. Targeting well-being through self-care actions [15], such as making positive lifestyle choices, is essential for self-management.

Wearable devices, mobile apps, and smart home technologies can facilitate dementia care by providing education and improving self-care knowledge. They can improve sleep and air quality [16], encourage exercise, and promote dietary interventions such as the Mediterranean-DASH Diet Intervention for Neurodegenerative Delay diet [17]. These technologies can support health pathways for conditions such as hypertension [18], diabetes [19], depression [20], and hearing loss [21], which are known to predispose individuals to dementia. In summary, lifestyle interventions supported by technology, such as promoting physical activity, dietary improvements, cognitive stimulation, and social engagement, have consistently demonstrated effectiveness in reducing dementia risk [14], enhancing QoL, and mitigating dementia‑associated behaviors associated with disease progression.

### Aims and Objectives

This study aims to systematically review and critically appraise current literature evaluating the impact of wearables, mobile apps, and smart home technologies aimed at enhancing self-management, reducing dementia-associated behaviors, and improving QoL for people with dementia. It also seeks to assess how closely these interventions align with recognized care needs, guidelines, and health outcome standards. This review maps the current landscape of digital interventions against these care priorities to identify clearly defined care pathways, highlight gaps where pathways are insufficient or absent, and recommend areas requiring development or improvement.

### Research Questions

This systematic review applies the PICO (population, intervention, comparison, and outcomes) framework to evaluate wearable technology, smart home technologies, and mobile apps in dementia care. The following research questions guided the review:

1. Which key demographic groups were targeted by the intervention? (target demographics)
2. What does the digital or complex intervention consist of? (intervention details)
3. How effective were the interventions? Which QoL or dementia‑associated behavioral outcomes were targeted? How were these outcomes measured? How do they collectively map to established taxonomies? (outcome evaluation and effectiveness)
4. How accessible are the interventions for ongoing use in terms of availability, cost, and ease of use? (intervention accessibility)
5. What methodologies and study designs were used to evaluate the efficacy of the interventions? How were biases and confounding factors addressed? (study design quality)

The 5 subheadings are reflected in the Results and Discussion sections to facilitate navigation.

## Methods

### Overview

The research questions were structured using the PICO framework. We preregistered the review protocol on PROSPERO (CRD42023461841). To develop the search strategy for this literature review, we consulted a professional librarian to ensure comprehensive and effective search methodologies. A search strategy was established to identify relevant publications using keywords to identify the target disease types (Multimedia Appendix 1). Briefly, search terms included “quality of life,” “dementia,” various technology-related terms (eg, “wearable technology,” “health technology,” and “gerontechnology”), and “outcome measurement.” Our search focused on changes in QoL and dementia‑associated behaviors, as measured by validated assessments.

### Eligibility Criteria

The inclusion and exclusion criteria are presented in Textbox 1.

**Inclusion and exclusion criteria.**

**Inclusion criteria**

- Adults with common or mixed types of dementia, including Alzheimer disease, vascular dementia, frontotemporal dementia, and Lewy body dementia
- Participants with dementia using wearable technology, smart home technologies, or mobile apps, where the intervention demonstrated a measurable improvement in quality of life or addressed behavioral changes linked to cognitive decline and dementia while supporting dignity and autonomy
- Technology used for, or with clear potential for, self-management with or without caregiver assistance
- Care coordination interventions, only if they led to the empowerment or autonomy of users in the context of self-management
- Peer-reviewed studies published in English between January 1, 2013, and September 30, 2023, including randomized and nonrandomized controlled trials, observational studies, qualitative studies, and mixed methods studies

**Exclusion criteria**

- Studies that did not meet the inclusion criteria, as determined through the screening and review process (Multimedia Appendix 2)
- Diagnostic interventions without a clear self-management component for individuals with dementia
- Technologies designed primarily for use by carers or health professionals without direct engagement by the person with dementia
- Studies not subject to academic peer review (eg, gray literature, policy briefs, and short conference abstracts)

We limited the search to studies published between January 1, 2013, and September 30, 2023. The chosen starting year marked a significant shift in dementia care technology research due to 3 key technological advancements. First, smartwatches became prevalent in biomedical applications [22]. Second, consumer smart home hubs such as SmartThings, Google Home, Apple Home, and Amazon Echo gained widespread adoption, bringing “communication,” “living,” and “health” into focus as major research themes [23]. Finally, mature mobile app ecosystems greatly increased the availability and use of mobile health apps [24]. By contrast, earlier studies predominantly relied on now-obsolete technologies, including PDAs and pagers [25], limiting their relevance to contemporary dementia care practices. Extending the window through 2023 allowed the inclusion of >10 product cycles of smartphones and smartwatches as well as several iterations of smart home hubs, thereby ensuring technological relevance while allowing multiple device generations to be evaluated.

For the purposes of this review, “digital self-management interventions” were defined as technology-based tools that directly prompt or guide individuals with dementia, with the aim of improving QoL or reducing unwanted dementia-associated behaviors; for example, this may include a mobile phone reminder app that interacts directly with the person with dementia.

To ensure consistent and rigorous application of the eligibility criteria, the reviewers used a structured decision table in conjunction with the predefined search strategy (Multimedia Appendix 2).

### Search Strategy

The searches were conducted by a single author (GC) across the following databases: PubMed, Scopus, ACM Digital Library, CINAHL (via EBSCO), PsycInfo (via EBSCO), Web of Science, IEEE Xplore, Embase (via Ovid), and MEDLINE (via Ovid). Minor adjustments were made to the search syntax for each database to optimize results. Searches were limited to peer-reviewed studies published in English between January 1, 2013, and September 30, 2023 (Multimedia Appendix 1). The searches retrieved 1242 records, as follows: PubMed (n=302, 24.31%), Scopus (n=211, 16.99%), ACM Digital Library (n=20, 1.61%), CINAHL (n=153, 12.31%), PsycInfo (n=85, 6.84%), Web of Science (n=269, 21.66%), IEEE Xplore (n=161, 12.96%), Embase (n=7, 0.56%), and MEDLINE (n=34, 2.73%; Multimedia Appendix 1).

### Selection Process

We adhered to the PRISMA (Preferred Reporting Items for Systematic Reviews and Meta-Analyses) guidelines [26]. Records were first imported into reference management software (EndNote [Clarivate]) by a single reviewer (GC), and duplicates were subsequently removed.

Records were then imported into Rayyan (Rayyan Systems Inc), a web-based application designed to facilitate the systematic review process. Two reviewers (GC and WH) initially reviewed 50 abstracts together to establish consistent screening practices. Subsequently, they independently and blindly screened all abstracts using a decision table for guidance (Multimedia Appendix 2). Exclusions were marked with at least 1 exclusion reason from the table. The level of agreement between the reviewers was assessed using the Cohen κ coefficient (Multimedia Appendix 3) to ensure interrater reliability. A consensus meeting was then held to discuss individual reviews and resolve any disagreements, with a third reviewer (KE) available to provide additional reviews and facilitate consensus.

The full-text review process mirrored the abstract screening. The team first reviewed a small selection of full-text articles together to establish consistency. The two lead reviewers then independently conducted blinded full-text reviews, using the same decision table and recording exclusion reasons as applicable. Interrater agreement was again assessed using the Cohen κ coefficient (Multimedia Appendix 3), followed by a consensus meeting with the third reviewer. The included articles were subsequently analyzed, and relevant data were extracted, normalized, and stored in a custom XML database (eXist-db [eXist Solutions]), chosen for its flexibility in storing and grouping metadata.

### Extracted Data From Studies

#### Overview

Due to substantial heterogeneity in study designs, outcome measures, and data collection tools, a meta-analysis was not feasible. Instead, data were synthesized using a structured narrative synthesis and quantitative descriptive synthesis approach. Initial synthesis was informed by documented screening discussions between the two lead reviewers (GC and WC), who consolidated their notes and extracted data. A third reviewer was available to resolve any disagreements or discrepancies, guided by predefined inclusion and exclusion criteria (Textbox 1 and Multimedia Appendix 2). Themes were systematically identified, enumerated, and mapped against relevant care guidelines to facilitate interpretive comparisons. Methodological quality and risk of bias were assessed using established critical appraisal tools. One reviewer (GC) extracted all study data as notes into Rayyan. The same reviewer then conducted automated internal consistency checks, using multifile regular expression matching and batch analysis in a programmer’s text editor. A second reviewer (WH) independently verified every extraction, and any discrepancies were resolved through discussion with a third reviewer (KE), ensuring rigor, transparency, and consensus.

Custom branches of extracted metadata were organized into groups related to the study goals (including the hypothesis or evidence base), user demographics and health information, quality assessment metrics, user needs classification, user outcomes classification, QoL and dementia‑associated behavioral measurement classification, device use and accessibility, support groups for technology use, and user activities associated with technology use. This organization was intended to assist with carrying out a battery of quality measures and address the 5 research questions.

#### Evaluation Frameworks

##### The National Institute for Health and Care Excellence Quality Standard 184

The National Institute for Health and Care Excellence (NICE) quality standard (QS) 184 benchmarking tool was chosen as part of our assessment because it addresses the prevention of dementia, along with the assessment, management, and provision of health and social care support for individuals with dementia, outlining high‑quality care objectives to guide improvements in care delivery [27]. The standard was published in 2019, after many of the included studies were conducted, and is specific to England and Wales; therefore, most authors would not have been aware of it at the time of their research. However, the standard offers a valuable lens through which to interpret the findings, particularly by highlighting areas now recognized as especially important in the design and evaluation of dementia-related technologies. Within this framework of 7 quality statements, 5 statements directly pertaining to technology-based intervention were included. The remaining 2 statements—related to “diagnosis” and “coordinating care”—were excluded because they pertain primarily to health professionals’ responsibilities and fall outside the scope of this self-management–based intervention study, consistent with the inclusion and exclusion criteria presented in Textbox 1.

The 5 included QS 184 areas were as follows:

- Raising awareness—health promotion interventions
- Advance care planning
- Activities to promote well-being
- Managing distress
- Supporting carers

##### The Nursing Outcomes Classification

The Nursing Outcomes Classification (NOC) [28], developed by the University of Iowa, is a comprehensive and standardized classification system used to evaluate patient outcomes from nursing interventions. It includes 621 nursing outcomes designed to facilitate a thorough evaluation of care outcomes. A significant advantage of the NOC is its widespread integration into clinical practice and its incorporation within the internationally standardized Systemized Nomenclature of Medicine–Clinical Terms system [29], enabling interoperability across health care settings. This standardized approach promotes the consistent documentation, evaluation, and enhancement of patient care, making it the preferred framework for this systematic review, rather than alternative tools such as the International Consortium for Health Outcomes Measurement (ICHOM) set of patient-centered outcome measures for dementia or the World Health Organization dementia guidelines, both of which lack comparable integration and broader clinical implementation.

Given the specific focus of our research on dementia-related outcomes pertinent to QoL and dementia‑associated behaviors in dementia self-management, we further refined the extensive NOC list. From the original 621 outcomes, a precisely targeted subgroup, consisting of 135 (21.7%) relevant criteria, was identified to directly address our research questions.

##### The Pugh Matrix

When analyzing multicomponent interventions, it is useful to separate the effects of individual components [30]. This concept of decomposing complex interventions into simpler, measurable, and more comparable features allows for a clearer assessment. The Pugh matrix, also known as a decision matrix, is an evaluative framework for this purpose developed by Pugh [31] in the 1960s at the Unilever Research Laboratory and presented to the science community in the 1980s. It facilitates a structured method to break down, quantitatively assess, score, and compare metrics based on weighting that can be altered to amplify key parameters.

### Data Synthesis

Given the wide variation in study designs, intervention types, and outcome measures, we used a structured narrative synthesis rather than a meta-analysis. The synthesis proceeded in 4 steps, as described in the following subsections.

#### Domain-Based Grouping

Each study was mapped to the 5 outcome domains specified in our research questions: demographics, intervention details, QoL or dementia-associated behaviors, accessibility for self-management capacity, and study quality.

#### Critical Appraisal Integration

Methodological quality was evaluated with the relevant studies grouped by design type for the appropriate Critical Appraisal Skills Program (CASP) checklist [32] and the Mixed Methods Appraisal Tool (MMAT) assessment [33]. Studies were categorized as low, moderate, or high quality. These tools were selected to ensure a consistent, robust evaluation of methodological quality across diverse study designs, helping to identify potential sources of bias. During synthesis, findings from moderate- and high-quality studies were given greater interpretive weight.

#### Benchmarking Across Multiple Frameworks

A custom Pugh matrix feature comparison table was developed (Multimedia Appendix 4 [28,29,34-55]), and subgroup analyses using the NICE QS 184 quality statements (Multimedia Appendix 5 [28,29,34-55]) and the NOC (Multimedia Appendix 6 [28,29,34-55]) were conducted to benchmark each intervention against the following criteria:

- NICE QS 184 (high‑priority dementia care needs)
- NOC
- QoL and dementia‑associated behavioral outcome measurement indicators
- Study population and duration
- Intervention details and technology type
- Accessibility for self-management

#### Descriptive Aggregation and Visualization

Frequencies and proportions were calculated for the presence and direction of effects within each domain. The results are presented in an integrated study quality and bias table and illustrated with supporting visuals (eg, technology iconography, a world map of study locations, and radial plots of outcome alignment).

## Results

### Overview

**Figure 1:**
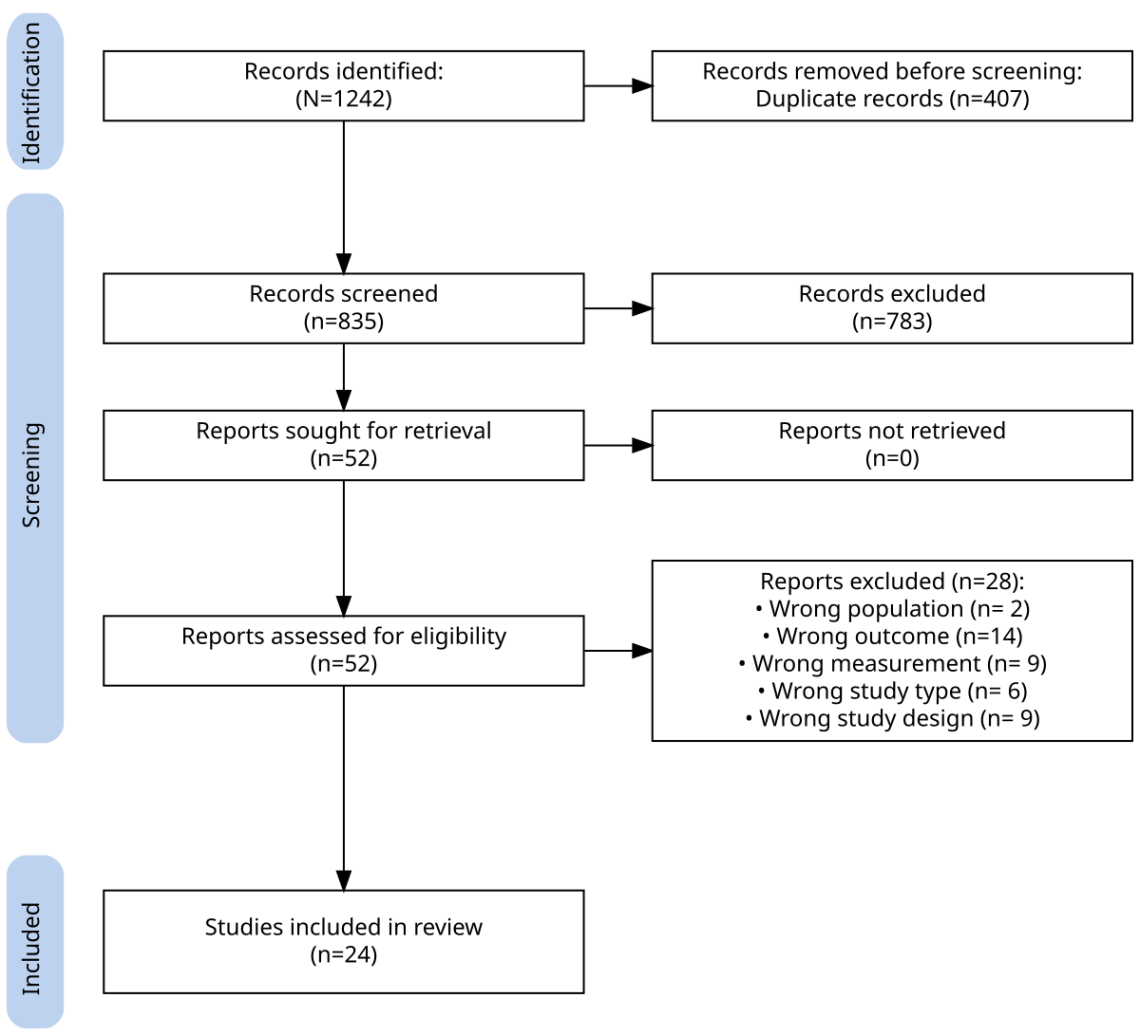
PRISMA (Preferred Reporting Items for Systematic Reviews and Meta-Analyses) flow diagram. Several studies met >1 exclusion criterion; consequently, the summed counts of exclusion reasons exceed the 28 studies excluded at full-text screening.

Our search across 9 databases for studies published over a 10-year period yielded 1242 records. From these 1242 studies, we removed 407 (32.77%) duplicates, leaving 835 studies (67.23%) for screening. We excluded 783 (93.8%) of these 835 studies during initial screening, leaving 52 (6.3%) for full-text assessment, of which 24 (46%) were included in the analysis (Figure 1).

Of the 835 articles originally screened, both reviewers unanimously excluded 720 (86.2%) and included 39 (4.7%); the remaining 76 (9.1%), initially flagged as “maybe,” were evaluated with a third reviewer during a consensus meeting. Interrater agreement, assessed using the Cohen κ coefficient, yielded a score of 0.472, indicating moderate agreement that exceeded chance. Full details of the screening process and κ calculations are provided in Multimedia Appendix 3.

During a second screening phase, 76 articles flagged by only one reviewer were re-examined. Many (25/52, 48%) were excluded for mentioning dementia only tangentially or failing to assess relevant outcomes, leading to the unanimous exclusion of 783 (93.8%) of the 835 articles in total. A further 52 articles remained for full-text screening, which each reviewer then assessed independently and blindly. Cohen κ was calculated as 0.383, reflecting a fair level of agreement before discussion; however, perfect consensus was ultimately reached. Full details, including reasons for inclusion and exclusion, are provided in Multimedia Appendix 3.

This review systematically examined the current evidence on digital technologies, including wearables, mobile apps, and smart home systems, that are designed to support self-management, mitigate dementia-associated behaviors, and enhance QoL for people living with dementia. The findings are presented according to the 5 predefined research questions and are structured to evaluate the impact of these interventions, their relevance to recognized care needs, and their alignment with care guidelines and an established health outcome standard.

### Target Demographics

The majority of the included studies (18/24, 75%) did not report specific participant numbers or dementia subtypes. Of the studies that did provide relevant data, the majority (4/6, 67%) focused primarily on individuals with Alzheimer disease. Specifically, 81 (88%) of the total 92 participants were identified as having Alzheimer disease; studies on atypical dementias were scarce. Of the total 92 participants, 5 (5%) were identified with vascular dementia, 2 (2%) with Lewy body dementia, and 4 (4%) with mixed dementia; none had frontotemporal dementia (Multimedia Appendix 4).

Target demographics are important because a range of sociodemographic factors can influence dementia-related diseases. These factors include environmental exposures [56], genetic predispositions [57], lifestyle choices [58], and access to health care services [59], all of which can impact study outcomes in addition to the study interventions themselves.

Age is the primary factor influencing dementia risk [1]. Participant ages across the studies ranged from 40 to 97 years, with most of the studies (13/24, 54%) reporting participants in their 70s or 80s; the most commonly reported average age was in the early 80s. Gender, a significant risk factor [60], was generally reported and generally intentionally balanced in most of the studies (16/24, 67%), with a slightly higher proportion of women overall, possibly reflecting their higher likelihood of developing Alzheimer disease and their longer life expectancy.

Educational level (measured as years of schooling) was reported in approximately half of the studies (11/24, 47%). A small number of studies also reported participants’ smoking status (4/24, 17%), drinking status (4/24, 17%), and past occupation (3/24, 13%).

**Figure 2:**
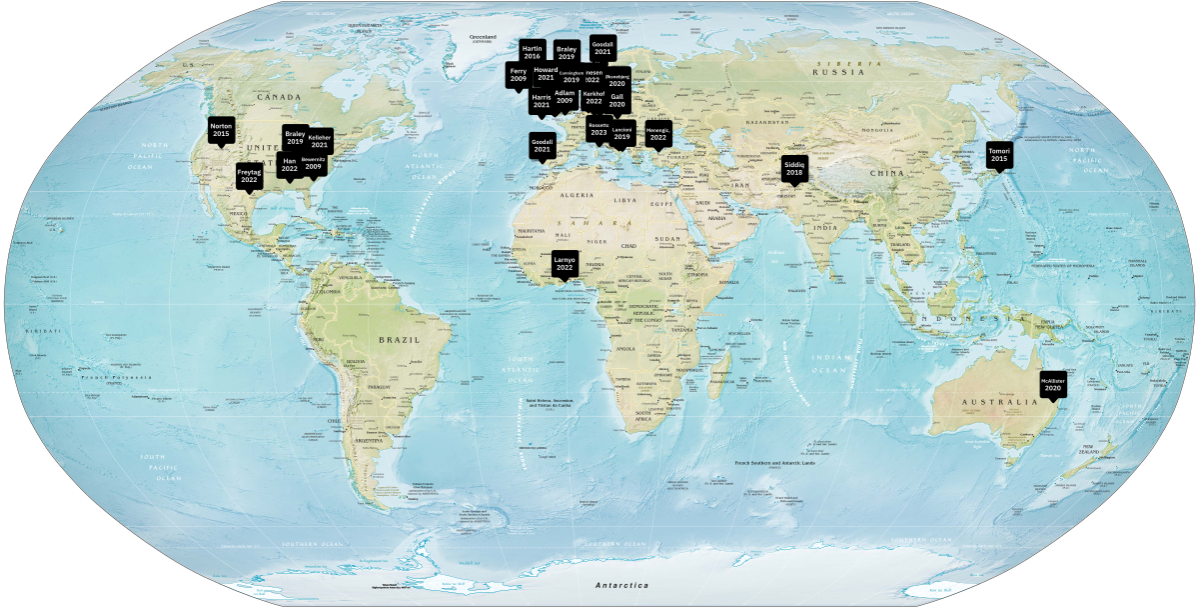
Geographic distribution of studies.

Most of the studies (18/24, 75%) did not report the race of participants. Among those that did, participants were primarily White and English speaking, mostly from Western countries (Figure 2 [28,29,34-55]). Only a few studies (3/24, 13%) included racial and ethnic minority individuals. The studies were conducted across 24 locations: the United Kingdom (n=6, 25%), the United States (n=6, 25%), Denmark (n=2, 8%), Italy (n=2, 8%), Australia (n=1, 4%), Germany (n=1, 4%), Ghana (n=1, 4%), Japan (n=1, 4%), the Netherlands (n=1, 4%), Pakistan (n=1, 4%), Portugal (n=1, 4%), Norway (n=1, 4%), and Turkey (n=1, 4%; Figure 2).

**Figure 3:**
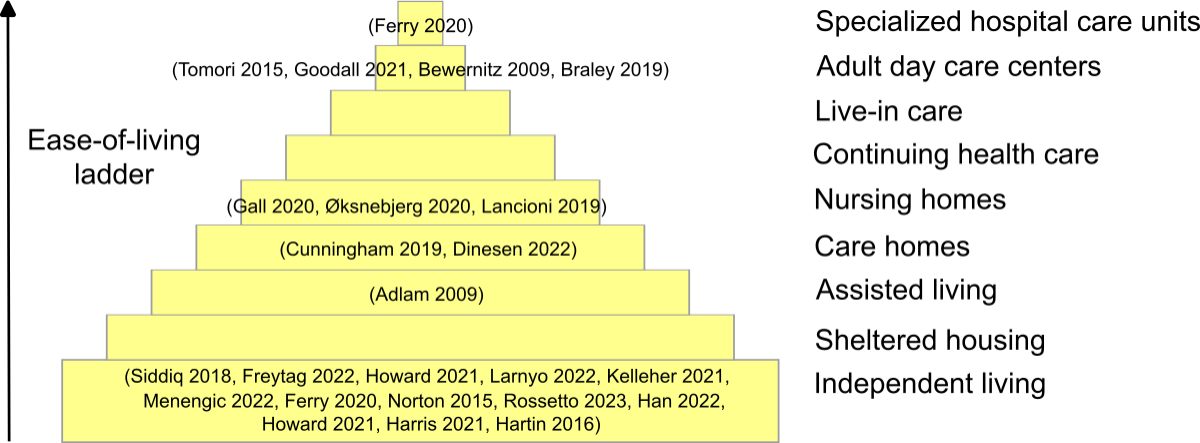
Environmental support for daily living.

The 24 studies included people in the following types of environments, using terminology from the UK housing system: independent living (n=13, 54%), adult day care centers (n=4, 17%), nursing homes (n=3, 12%), care homes (n=2, 8%), specialized hospital care units (n=1; 4%), and assisted living (n=1, 4%); none included participants in live-in care, continuing health care, or sheltered housing (Figure 3 [28,29,34-55]).

### Intervention Details

From the 24 studies, we identified 9 main technology types used in the interventions. Of the 24 studies, 6 (25%) [34-39] included >1 type of technology as part of a complex intervention. Overall, the interventions included 13 wearables (multiple smart watches [34,35,37,38], multiple smart pendants [35,36,40], a blood pressure monitor [34], and the GPS Smart Sole [35]), 7 smart home technologies (the LOVOT social robot [41], multiple home activity sensors [36,39], near-field communication object triggers [37], and stimuli actuators [42]), and 19 mobile apps [28,29,34,37-39,43-55]. These interventions were intended primarily for use by people with dementia and their carers. The identified technology categories included smart home devices, personal devices (such as mobile phones and tablets), room- or community-based information screens, assistive robots, and vital signs monitoring devices. All technologies had the potential to be actively used by individuals with dementia for self-management. However, some systems required specialist installation or relied on passive elements, such as environmental monitoring via smart home devices and physiological tracking via wearables, to provide actionable decision support information that could be used or potentially used by people with dementia.

We collated the information extracted from the articles into a Pugh matrix for evaluation (Multimedia Appendix 4). The matrix synthesizes key aspects of intervention details, target demographics, outcome evaluation methods, and study design quality, limited to parameters that could be uniformly scored and compared. The studies varied widely in participant numbers (ranging from <10 to >700) and study durations (from single-day interventions to year-long assessments). The matrix revealed that some of the studies (6/24, 25%) showed exceptional results in specific areas but lacked consistency across multiple key factors required for a comprehensive and reliable evaluation; for example, the study on wearables in Ghana [35] scored highly due to its large sample size but performed less well in terms of study duration and the use of validated quality measures.

**Figure 4:**
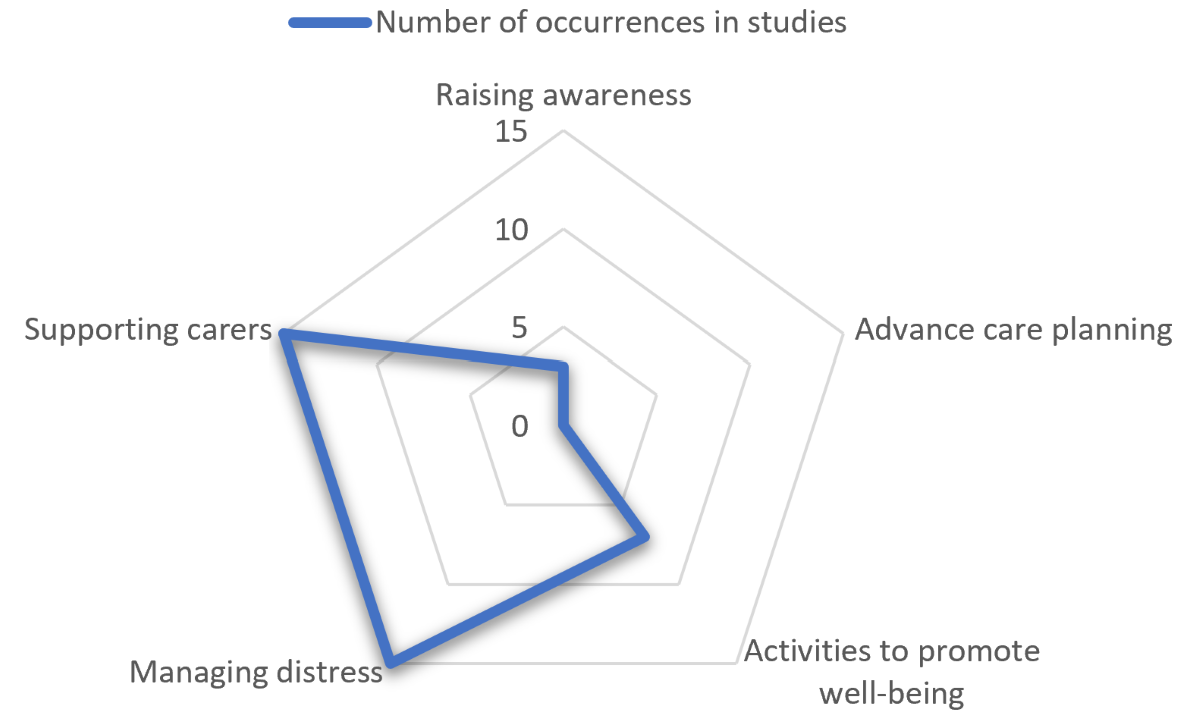
Analysis of all included studies against National Institute for Health and Care Excellence quality standard 184 quality statements 1, 3, 5, 6, and 7.

Each of the 24 selected studies was evaluated against 5 NICE QS 184 quality criteria to assess alignment with high-priority dementia care needs (Figure 4). The main areas covered were supporting carers (15/24, 62%) and managing distress (15/24, 62%). Notably, none of the studies addressed advanced care planning, although a few (7/24, 29%) included goal setting and activities, which are integral components of care planning. Only 3 (12%) of the 24 studies [38,52,53] emphasized the importance of raising participants’ awareness of, for example, midlife behavioral changes, which is crucial because nearly half of cases of dementia can be influenced by lifestyle factors that impact disease progression. Of the 24 studies, 6 (25%) [28,29,34-36,41] did not directly or indirectly target any of the areas covered in the QS 184 quality statements. Multimedia Appendix 5 presents a study-by-study subgroup analysis based on the NICE QS 184 quality statements.

### Outcome Evaluation and Effectiveness

Considerable heterogeneity across study designs precluded the planned quantitative synthesis. Most of the included papers (20/24, 83%) were small pilot studies characterized by incomplete reporting and unblinded outcome assessment. Only 6 (25%) of the 24 studies were randomized controlled trials (RCTs) [28,29,34,36,38,53]; the remainder comprised qualitative studies or uncontrolled feasibility studies. Effect‑size estimates were reported in just 3 (13%) of the 24 studies (none provided CIs), and only 1 (4%) of the 24 trials addressed QoL or dementia‑associated behavioral outcomes [36]. This trial, which was methodologically robust, demonstrated a small but statistically significant decline in QoL at 24 months. Given these limitations, the overall effectiveness of the interventions remains uncertain, and the quantitative pooling of QoL or dementia‑associated behavioral data was not feasible. Accordingly, we proceeded with a structural narrative synthesis, detailed in the following paragraphs.

**Figure 5:**
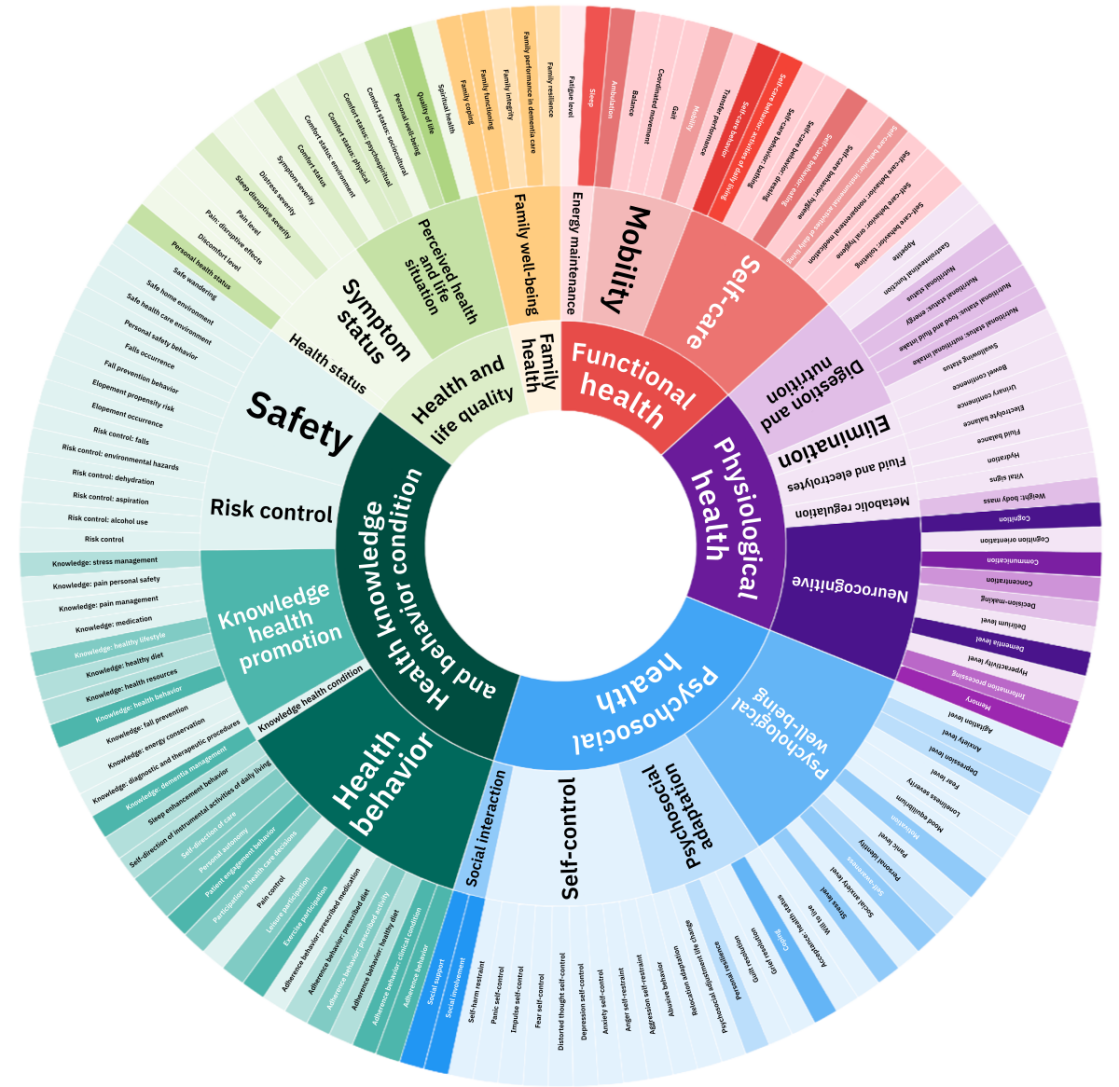
Combined context of study outcomes based on Nursing Outcomes Classification subgroups. The 3 concentric layers represent domains, classes, and subclasses. Hue intensity denotes article volume: the darkest signifies most articles, while the palest signifies none. Domain color coding is as follows: functional health (red), physiological health (purple), psychosocial health (blue), health knowledge and dementia‑associated behavior condition (dark green), health and life quality (pale green), and family health (orange).

**Figure 6:**
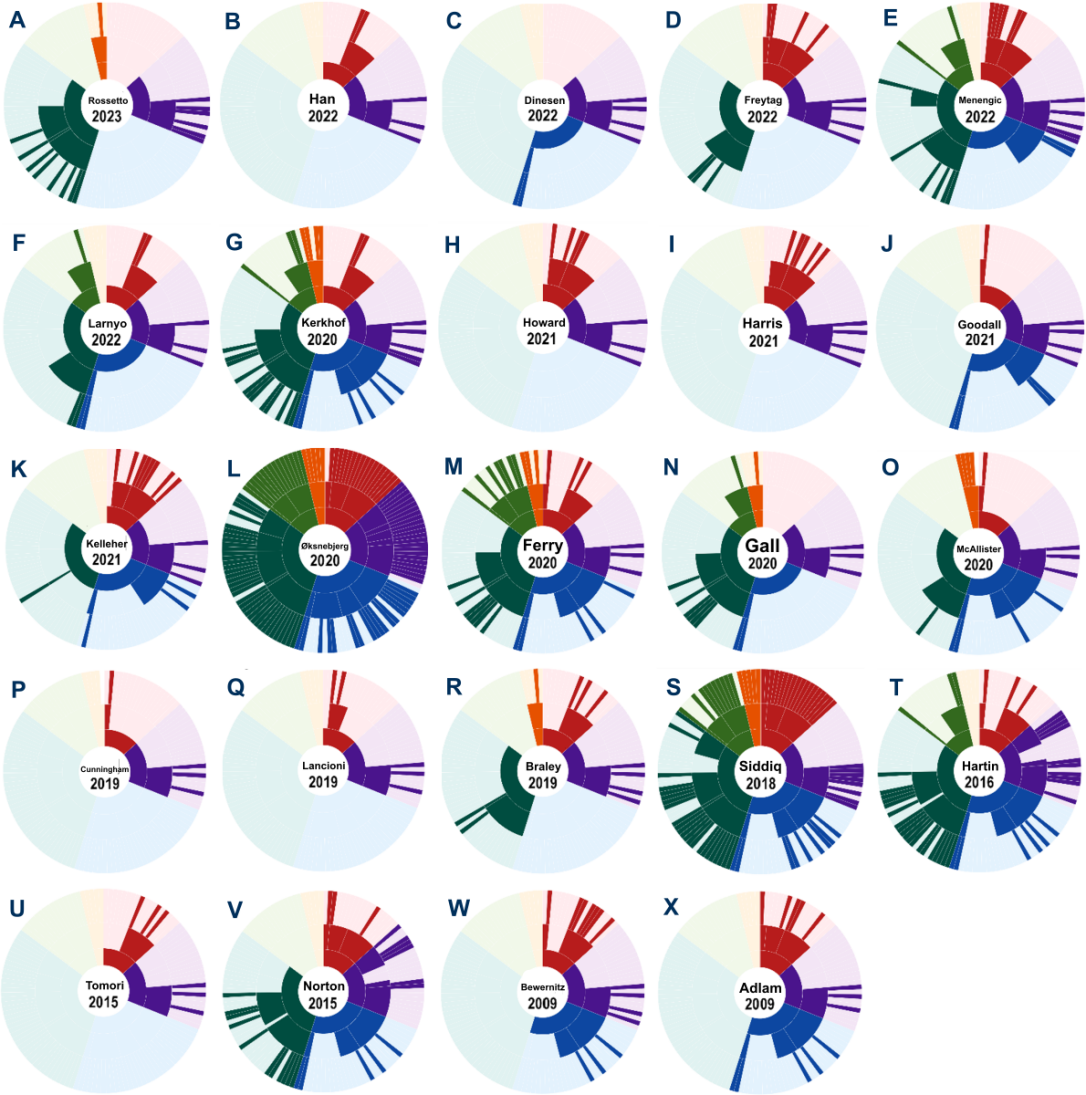
Individual study outcomes based on Nursing Outcomes Classification subgroups. The 3 concentric layers represent domains, classes, and subclasses. Hue intensity denotes article volume: the darkest signifies most articles, while the palest signifies none. Domain color coding is as follows: functional health (red), physiological health (purple), psychosocial health (blue), health knowledge and dementia‑associated behavior condition (dark green), health and life quality (pale green), and family health (orange).

Across the 24 included studies, most (n=19, 79%) targeted a narrow range of outcomes, primarily neurocognitive, health behavior, and self-care (Figure 5). However, a few studies (5/24, 21%) [29,47,48,52,53] addressed a broader range of outcomes (Figure 6 [28,29,34-55]).

Figure 5 illustrates the broader NOC domains, their classes, and outcomes with slices distinguished by color. The darker the color (the stronger the hue), the more frequently the domain, class, or outcome was referenced in the 24 studies. The lightest color indicates concepts not represented in any study.

Figure 6 presents a study-by-study mapping of the specific outcome themes addressed in each of the 24 studies.

The Pugh matrix feature comparison table (Multimedia Appendix 4) shows the diverse and distinct quality differences in the measurement instruments used across the 24 included studies to assess QoL and dementia-associated behaviors. We had set out to identify validated measures, such as the Quality of Life–Alzheimer’s Disease Scale [61], and behavioral changes related to cognitive decline and dementia measured by assessments such as the Revised Memory and Behavior Problems Checklist [62], but the results revealed a much broader range of measurements.

### Intervention Accessibility

We appraised accessibility based on cost, availability, and ease of use for people with dementia. The included studies used a variety of devices, ranging from lower-cost items such as the Nike+ FuelBand (US $149 at the time of the study) to higher-cost devices such as the LOVOT robot (US $3000 at the time of the study). In addition, most of the studies (14/24, 58%) used mobile phones or tablets (at the time of reporting, the most popular Apple iPad Air tablet was available for US $549 on the Apple website).

The devices used across the studies were predominantly consumer electronics, typically available from multiple manufacturers and subject to regular updates and improvements. Regarding their accessibility, most were not specifically marketed for dementia care and required complex configurations or developmental modifications for integration into care interventions. These specialized adaptations limit accessibility for end users.

**Figure 7:**
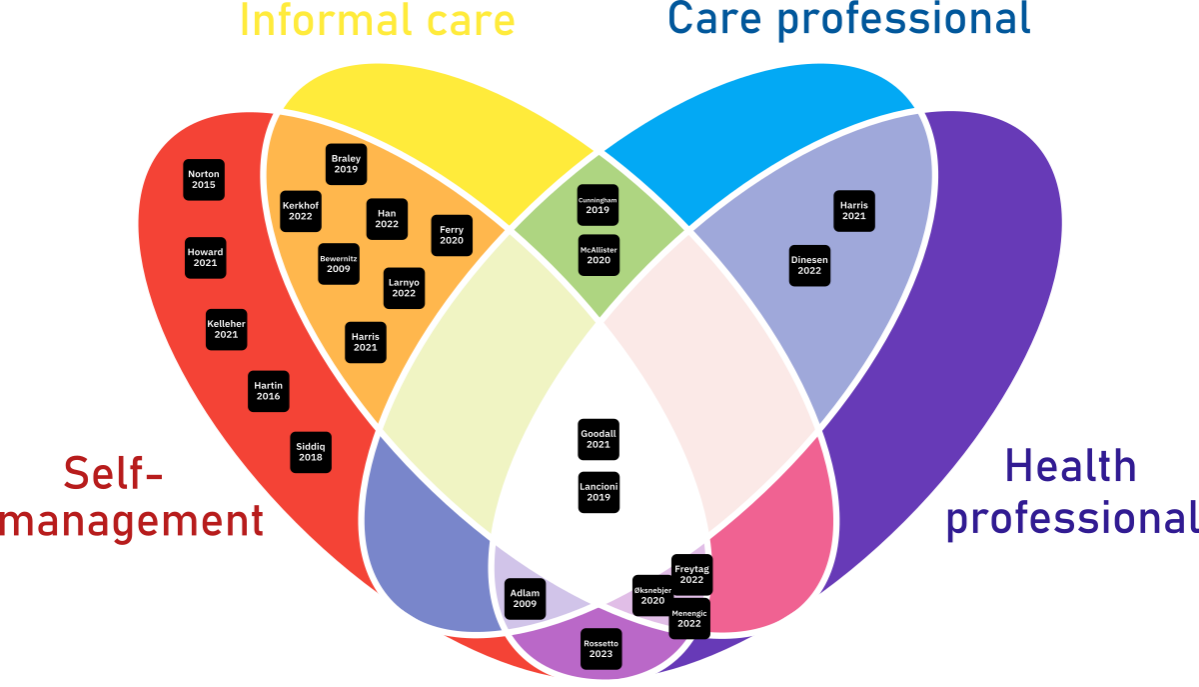
Circle-of-care user groups represented in the studies.

The studies were based on circle-of-care user groups, as illustrated in Figure 7 [28,29,34-55]. More than half of the studies (19/24, 79%) focused on self-management, which was also supported by informal carers, and only 5 (21%) of the 24 studies examined self-management technology that was not supported by any health or care group. Although several studies (14/24, 58%) identified informal carers or focused on dyads (a person with dementia and their primary caregiver), most (11/14, 79%) did not quantify the level of informal care support available to participants, which could significantly influence the results.

### Study Design Quality

To rigorously evaluate each included study’s methodological quality, we used 3 tools: the MMAT, the CASP checklists, and a custom characterization table developed specifically for this review. Each study was independently assessed by two reviewers; disagreements were resolved through consensus or by consulting a third reviewer. The following paragraphs briefly describe these tools and outline the approach used to identify and address the risks of bias.

The Pugh matrix table (Multimedia Appendix 4) highlights higher-quality outcome measurements in several of the studies (4/24, 17%) [28,29,39,48], making them easy to identify. The table additionally highlights the quality of studies that included more participants for a longer duration. The assistive technology and telecare in supporting independent living study [36], received a high score due to its large number of participants; however, its narrow outcome focus may reduce its impact and applicability.

All studies were appraised for quality using the MMAT and were rated as either good or excellent, as they met most or all quality criteria (Multimedia Appendix 7 [28,29,34-55]).

We also conducted a CASP quality assessment to address the diverse range of study types (Multimedia Appendix 8 [28,29,34-55]), supplementing the MMAT assessment of mixed methods studies. The CASP assessment indicated excellent overall quality in the qualitative studies, good quality in the cohort studies, and areas for improvement in the RCTs. Specifically, the concerns with the RCTs included a lack of blinding and limited generalizability due to demographic specificity, which may affect reproducibility.

This review used a rigorous methodology, including a comprehensive literature search, clearly defined inclusion and exclusion criteria, structured data extraction, and systematic application of the CASP checklists. Findings from the CASP assessments were integrated with the MMAT results and a custom-developed characterization table to categorize studies by overall risk of bias (low, moderate, or high). This integrated assessment approach informed subsequent decisions on data synthesis and the weighting of study conclusions, ensuring that the final interpretations accurately reflected the quality and robustness of the evidence base (Table 1).

**Table 1:** Summary of integrated study quality and risk-of-bias assessment.

Study	Study design	MMA^a^ summary (criteria met; %^b^)	CASP^c^ summary assessment (%)	Key observations from custom table notes	Issues assessment	Score	Overall risk of bias^d^
Menengiç et al [28]	RCT^e^	87.5	81	6-wk exercise study (n=10; Alzheimer disease): limited scope but comprehensive outcome assessment	Low	22.6	Moderate
Norton et al [38]	RCT	90	53	5-mo smartphone study with older activity-monitoring technology: limited outcomes reported	Moderate	18.5	Moderate
Rossetto et al [34]	RCT	90	76	6-wk study (n=21; Alzheimer disease): focused on QoL^f^ only; no behavioral data; limited self-management (medical expertise needed for the Ability telerehabilitation program)	Moderate	18.15	Moderate
Howard et al [36]	RCT	80	62	2-y study (n=248) of assistive technology: minimal detail on the intervention but high-quality, multioutcome assessment	Moderate	56.2	Moderate
Hartin et al [53]	RCT	80	69	5-mo study (n=116; 21 with Alzheimer disease): limited behavioral outcomes, no QoL assessment, and reliance on outdated technology	Moderate	23.15	Moderate
Gall et al [46]	Qualitative study	100	90	6-mo study (n=8) with the CareShare app: narrow carer focus; limited outcomes, lacking standard QoL or behavioral measures	Moderate	14.15	Low
Goodall et al [42]	Qualitative study	100	100	3-mo study (n=7) evaluating a sensory garden: limited outcomes without standard QoL or behavioral measures	Moderate	8.35	Low
Kerkhof et al [29]	RCT	100	100	3-mo study (n=7) of FindMyApps: rich detail but serves mainly as a tool to locate interventions; good QoL and behavioral assessments	Moderate	31.1	Low
Braley et al [51]	Qualitative study	100	100	Single-d test (n=15) of smart home autoprompting: observed daily living via video only, not integrated with self-management	Moderate	5.3	Low
Cunningham et al [50]	Mixed methods study (qualitative research)	75	90	2-wk study (n=14) using music reminiscence: task prompts via various triggers; good QoL and behavioral outcome assessments	Moderate	9.2	Low
McAllister et al [49]	Qualitative study	100	90	6-mo study (n=3) with the Memory Keeper app: personalized prompts; usability and impact assessed rather than QoL or behaviors	Low	14.1	Low
Dinesen et al [41]	Mixed methods study (qualitative research)	87.5	100	3-mo study (n=43; 20 with Alzheimer disease) using the LOVOT robot: limited outcomes focused on acceptability and basic behaviors	Moderate	21.5	Low
Øksnebjerg et al [48]	Qualitative study (cohort study)	100	73	3-mo study (n=19) with the ReACT app: strong goal-setting, self-management, and cognitive metrics; high-quality QoL and behavioral assessments	Low	35.7	Moderate
Bewernitz et al [55]	Qualitative study (cohort study)	100	85	11-participant study conducted on 1 d; limited prompting efficacy on a subgroup of users with only 3 activities of daily living	Moderate	5.4	Low
Ferry et al [47]	Quantitative descriptive study (cohort study)	80	65	3-mo study (n=30) of the InspireD reminiscence app: QoL assessed but no self-management or behavioral measures	Moderate	17.65	Moderate
Harris et al [45]	Qualitative study (cohort study)	100	81	6-mo study (n=96) using the Gray Matters mobile app: robust self-management and behavioral outcomes; no QoL measures reported	Low	23.15	Low
Lancioni et al [37]	Quantitative descriptive study (cohort study)	90	81	2-d study (n=21): smartphone-based cognitive stimulation for arm movement in 2 activities; limited self-management relevance, obsolete technology, lacking QoL and full behavioral data	Moderate	4.15	Low
Han et al [43]	Qualitative study (cohort study)	80	88	6-wk study (n=7) with visual mapping assistive technology: focus on carer burden reduction rather than self-management; good behavioral assessment, minimal QoL data	Moderate	17.45	Low
Kelleher et al [44]	Nonrandomized study (cohort study)	90	77	3-mo study (n=14) using the MapHabit app: limited outcomes (mainly behavioral acceptability) for a narrow set of daily tasks	Moderate	15.25	Low
Tomori et al [54]	Qualitative study (cohort study)	90	73	3-mo study (n=116; 21 with Alzheimer disease): strong self-management and accessibility coverage but primarily cognition focused, lacking QoL and behavioral measures	Moderate	14.55	Moderate
Adlam et al [39]	Quantitative descriptive study (cohort study)	90	96	1-y study in a few sheltered “smart flats”: limited context, assessing behavioral outcomes only; no QoL data	Moderate	31.65	Low
Larnyo et al [35]	Quantitative descriptive study (cohort study)	90	69	Short survey (n=262) on health care wearables: limited follow-up; behavioral and minimal QoL outcomes assessed	Moderate	28.35	Moderate
Freytag et al [40]	Qualitative study (cohort study)	100	81	4-mo study (n=19): integrated wearable sensor data into clinical care; comprehensive goal monitoring via surveys and sensor data	Low	20.25	Low
Siddiq et al [52]	Qualitative study (cohort study)	100	73	Short test (n=25) of the CareD mobile app: focused on daily assistance and self-management; limited outcome scope, mainly app acceptability	Moderate	17.9	Moderate

^a^MMAT: Mixed Methods Appraisal Tool.

^b^The proportion of compliant criteria; this is not a quality score or rating.

^c^CASP: Critical Appraisal Skills Program.

^d^Risk of bias was categorized as follows: MMAT—low (≥90%), moderate (75%-89%), and high (<75%); CASP—low (≥85% items addressed), moderate (65%-84%), and high (<65% or major concerns); custom table (Multimedia Appendix 4)—low (no critical issues), moderate (minor inconsistencies), and high (major design and reporting gaps); overall—low (all low or 1 moderate concern with strong quality), moderate (at least 1 moderate concern and no high concerns), and high (any high or ≥2 moderate concerns).

^e^RCT: randomized controlled trial.

^f^QoL: quality of life.

## Discussion

### Principal Findings

In this study, we undertook a systematic review to identify, compile, and appraise current literature on digital technologies, including wearables, smart home devices, and apps, designed to enhance self-management, reduce dementia-associated behaviors, and improve QoL for individuals with dementia. Many of the included studies (10/24, 42%) relied on qualitative methodologies, and effectiveness was often uncertain, with only 1 (4%) of the 24 studies clearly identifying a moderate decline in QoL at 24 months. Our key findings include the following:

- Interventions comprised 13 wearables, 7 smart home technologies, and 19 apps, primarily for people with dementia and their carers.
- Mapping to the NOC showed common outcomes: neurocognition (24/24, 100%), self-care measures (17/24, 71%), and health behavior (13/24, 54%). Needs benchmarking revealed common addressed needs, such as managing distress (15/24, 62%) and supporting carers (15/24, 62%).
- Despite the use of widely available technologies (eg, activity monitors, health care wearables, and digital prompters), accessibility was poor due to complex configurations and support needs.
- Overall, study quality ranged from good to excellent, with good-quality RCTs (n=6) and excellent qualitative studies.

We reviewed articles published over the past 10 years to assess the effectiveness of digital interventions for individuals with dementia, with an emphasis on self-management strategies aimed at enhancing QoL and mitigating adverse dementia‑associated behaviors associated with dementia. The included studies were systematically compared by mapping to standardized outcomes, QSs, and validated measures, normalizing variance to identify well-explored areas and gaps.

Our review found that most of the studies (18/24, 75%) focused on supporting health care professionals and carers, with only 5 (21%) of the 24 studies investigating unsupported technology used directly by patients with dementia. Alzheimer disease was the primary condition targeted in most of the studies (81/92, 88%). Notably, the quality of the studies was high, particularly the qualitative studies, which were excellent.

Most of the studies (14/24, 58%) involved the use of mobile phones or tablet devices. All included studies were distinct, with no evidence of chronological building on earlier findings within the study group. The diversity of dementia care technologies developed over the past 10 years reflects the extensive variety of individual needs and the complexity of dementia itself, necessitating highly customized solutions and making it challenging to adopt a unified approach. Technological fragmentation also arises from inconsistent funding models and short-term research grants favoring novel developments over incremental improvements. In addition, rapid technological obsolescence hampers continuous improvement and long-term collaboration on single solutions. Intellectual property restrictions further impede the sharing of knowledge and the ability to build on previous research. Collectively, these factors prevent researchers from effectively refining a single, widely adopted technology, resulting instead in numerous parallel developments tailored to specific needs or contexts.

The following subsubsections discuss these details further in relation to our 5 research questions.

### Target Demographics

Our analysis revealed a diverse range of sociodemographic factors and research objectives within dementia care, encompassing wearables, smart home systems, and mobile apps. The absence of key demographic information, such as race, educational level, and hearing loss in several of the included studies (21/24, 88%) limits this review’s ability to comprehensively evaluate the effectiveness of technology interventions for dementia. These demographic factors are recognized as important modifiers of dementia risk and progression, and when not measured or clearly reported, they may bias the findings or reduce their reliability. Consequently, future research must consistently record and report these demographic factors to enhance result validity and support the accurate assessment of technology interventions.

It was concerning that most of the studies (18/24, 75%) did not report participants’ racial backgrounds, particularly because racial discrimination in midlife has been linked to Alzheimer disease pathology in later life [63]. In addition, race markedly influences dementia risk, with African American individuals being twice as likely and Hispanic individuals one-and-a-half times more likely to develop Alzheimer disease than White Americans [64]. Research also indicates that tailored activities significantly reduce agitation and aggression behaviors, with an interaction effect showing greater reductions across different racial groups [65]. This underscores the importance of accounting for cultural and personal preferences, as they can significantly influence study outcomes.

It was disappointing that most of the studies (21/24, 88%) failed to report on hearing loss and other sensory impairments, which can significantly impact disease pathways; for instance, hearing loss alone can increase dementia risk by 2 to 5 times, depending on severity [66]. Similarly, it was concerning that educational level, a known modifier of disease risk [13], was not reported more frequently.

Apathy, an early indicator of dementia that may influence both decline and intervention outcomes, was also not assessed in any of the studies. This could have been quantified using a validated instrument such as the Apathy Evaluation Scale.

Most of the studies (21/24, 88%) could have improved results by meticulously controlling both nonmodifiable and modifiable factors associated with dementia progression. Standardizing methodologies and assessments for these variables increases research reliability and facilitates subsequent investigations. This approach enables data aggregation across multiple studies, enhancing knowledge synthesis and providing a robust foundation for future research.

The overrepresentation of studies (12/24, 50%) from the United Kingdom and the United States, despite most of the global population residing in Asia, likely stems from the inclusion of only English-language articles. This selection criterion is a limitation because it has presumably skewed the results toward English-speaking research centers.

### Intervention Details

It is noteworthy that many of the studies (4/24, 17%) that scored the highest on the Pugh matrix (Multimedia Appendix 4) exhibited weaknesses in specific areas. This suggests that while high standards are achievable across most measures, individual studies often fall short in certain aspects, indicating significant room for improvement even among the top performers.

It is notable that none of the studies aligned with the care standard guidance for supporting advanced care planning (Figure 4), despite its identification in the NICE QS 184 quality statements as one of the most important high-quality care objectives. The guidance recommends that “People with dementia and people involved in their care are supported to plan ahead and make decisions about future care when dementia is diagnosed and every time they have a care review” [67]. The health informatics standard ISO 13940 identifies a care plan as one that is relevant to self-management and defines it as a “dynamic, personalized plan including identified needed healthcare activity, health objectives and health care goals, relating to one or more specified health issues in a healthcare process” [68]. In addition, none of the studies addressed the NICE QS 184 guidance on raising awareness about lifestyle changes that can modify disease progression in dementia (Figure 4). Specifically, the guidance states that “People taking part in health and well-being programmes in midlife are told about lifestyle changes” [67]. This oversight is likely due to the significant time gap between midlife interventions and the typical onset of dementia in late life, along with the rapid obsolescence of consumer technologies used in these studies, which are often updated annually by manufacturers. Of the 24 studies, 6 (25%) [28,29,34-36,41] did not adhere to any of the NICE Quality Standard 184. They were prescriptive, focusing on testing specific exercises, activities, apps, home and wearables, or robots rather than on patient outcomes. These studies failed to support user choice, were not tailored to user needs and preferences, and did not encourage changes or recommend that people with dementia undergo a structured assessment before starting any intervention [67]. The studies used various commonly available sensing and interface technologies, innovatively adapted for dementia care. While some of the studies (3/24, 13%), such as the one by Dinesen et al [41] (LOVOT social robot), incorporated artificial intelligence and machine learning, none used large language models such as OpenAI’s ChatGPT and Google’s Gemini or a digital assistant such as Amazon’s Alexa. This omission likely reflects the inaccessibility of these technologies to researchers during the study period, despite their potential utility in digital assistants and in simplifying complex information for individuals with cognitive impairment.

### Outcome Evaluation Methods

It is impossible to measure expectation and placebo effects through standard scientific practices such as blinding and control in self-management–based technology interventions, as these involve direct human-computer interaction. Positive, health-reinforcing technology interventions that aim to support alignment with health indicators cannot be assessed as easily as binary outcomes (eg, hot or cold); instead, they often require longitudinal data collected over extended periods, which makes it very challenging to measure effectiveness in the short follow-up periods typical of most scientific studies. Diverse metrics, outcome heterogeneity, and a lack of consensus on usability and user experience measures [69], along with variation in individualized support, delivery, and training, also complicate the evaluation of effectiveness [70]. In addition, a significant problem exists in aligning user needs with intervention evaluation. People with dementia are on a journey in which both functional and intrinsic capacity decline over time and may fluctuate based on environmental influences, life events, and daily routines. These contextual changes make it very difficult to study effect sizes reliably [71].

**Figure 8:**
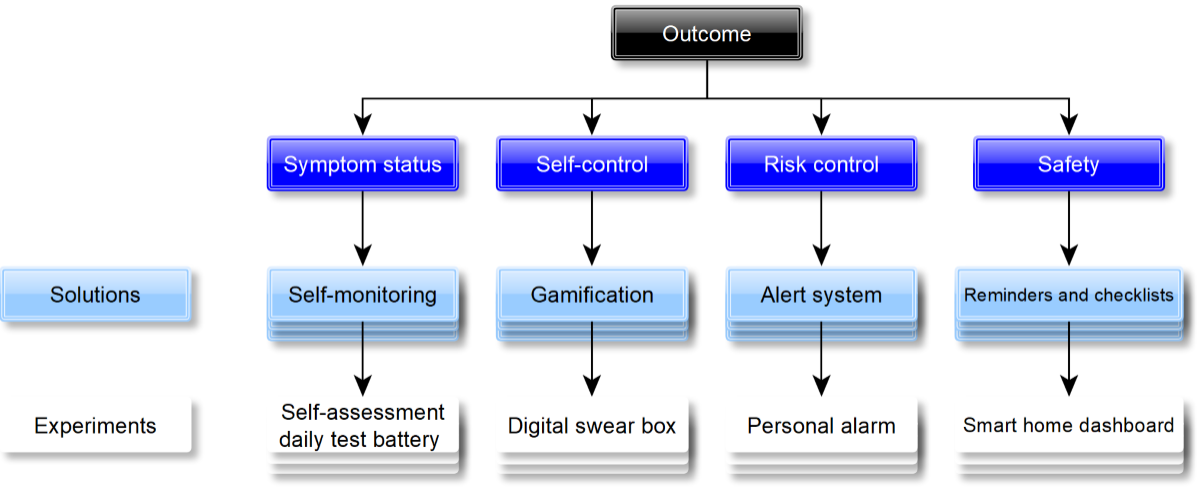
An example of a Nursing Outcomes Classification–based opportunity solution tree.

The NOC classification analysis (Figure 5) highlights some outcomes that were not well addressed by the included studies. These gaps include symptom status, self-control, risk control, and safety (Figure 8). This information could be used to guide the development of new solutions and experiments aimed at addressing the unmet needs of patients with dementia.

The CareD study [52] identified a significant limitation for many people with dementia: the need to download separate apps for each specific problem. Consequently, interventions should ideally encompass a wide range of functions to avoid overwhelming users. The widest coverage of NOC outcomes was achieved through mobile apps and goal setting [48,52], which offer multiple benefits for users while reducing the burden associated with learning a new app for every new problem. Another benefit of broad-coverage interventions is that they can be used by a wider patient demographic, as they can be adapted to specific individual and cultural needs.

We hypothesized that interventions would develop progressively, increasing in complexity and scope over the 10-year study period. We expected an evolution from specific, targeted approaches, such as the Aid for Decision-Making in Occupation Choice iPad app [54], toward more comprehensive, multifaceted interventions. These broader interventions, or “dementia super apps,” such as CareD [52] and ReACT [48], were anticipated to provide a wide range of services, embodying the concept of “super interventions.” However, our analysis did not reveal a discernible trend toward expanding outcome criteria over time (Figure 6), nor did it show that newer studies were systematically building upon the findings from earlier studies in the same areas.

The Pugh matrix table (Multimedia Appendix 4) shows that a broad range of measurement instruments were used. It is concerning that so many different methodologies exist for evaluating digital interventions, particularly those addressing identical user needs. There is potential to develop standardized test batteries for more uniform application, which could significantly advance research and scientific understanding in this field.

In this review focused on self-management, the Quality of Life–Alzheimer’s Disease Scale used in the Memory Tracks mobile app [50] was well suited due to its focus on practical aspects such as mood, memory, and relationships, as well as its dual format (self-report and proxy). We were disappointed that none of the selected articles focusing on dementia-associated behaviors used the Revised Memory and Behavior Problems Checklist, despite its comprehensive evaluation of both memory and dementia‑associated behavior problems essential for understanding the full scope of challenges faced by individuals with dementia. However, many of the studies (5/24, 21%) did use functional daily living assessments [34,36,40,43,45], such as the Bristol Activities of Daily Living Scale, and cognitive measures [28,40,45,48], such as the Addenbrooke Cognitive Examination and the Montreal Cognitive Assessment. These tools, when used in combination, are sufficient to validate a measure of dementia‑associated behavior change.

### Intervention Accessibility

We noted that many of the studies (4/24, 17%) focused on digital interventions that could only be used by carers or health care professionals; they could not be used by individuals with dementia for self-management. This finding aligns with other research noting a “lack of attention paid to the needs of end users, and subsequent tailoring of innovations to meet these needs” [72].

Simple, intuitive interfaces and targeted technology training are essential due to the generally limited digital literacy observed among older adults with dementia. The importance of tailored training is frequently highlighted in the literature; for example, Demiris et al [73] emphasized that older adults have varying functional limitations and prior technology experiences, necessitating personalized training approaches. However, despite the acknowledged importance of training, our review found that this aspect was notably underreported, with only 4 (17%) of the 24 included studies providing substantial details on training methods.

The InspireD feasibility study [47] notably allocated the most resources to user training, with training comprising more than half of the total intervention cost, almost twice the budget dedicated to software development. Similarly, the FindMyApps randomized controlled feasibility study [29] identified training as the most positively received component of the intervention, while the CareShare qualitative study [46] explicitly linked system adoption to the provision of comprehensive training. The Memory Keeper app study [49] reinforced these findings, highlighting that effective training significantly improved user confidence and reduced barriers to technology use.

By contrast, several of the studies (21/24, 88%) either minimally addressed or entirely overlooked user training; for instance, the study on a smart home autoprompting system [51] did not include formal user training in the intervention, although the discussion section acknowledged that early and adequate training might improve user comfort, familiarity, and trust. The study on tablet-based digital prompting [45] initially adopted an “out-of-the-box” approach without structured training or support but acknowledged that this approach was overly simplistic and ineffective. Conversely, the CareD study [52] reported persistent user difficulties despite implementing what was considered adequate training, suggesting that training quality and appropriateness also warrant careful consideration.

Many of the studies (21/24, 88%) evaluated the usability and accessibility of intervention interfaces, in some cases (9/24, 38%) using these factors as primary outcomes to validate their tools. Key considerations included reducing cognitive load through simplified navigation and memory support; ensuring inclusive designs that address visual, auditory, and motor limitations; and tailoring interfaces to individual cognitive profiles and preferences. Such personalization is crucial, given the heterogeneous manifestations of dementia. Notably, the Aid for Decision-Making in Occupation Choice iPad app [54] demonstrated one of the most accessible interfaces, effectively accommodating a range of functional and cognitive constraints.

Only 2 (8%) of the 24 included studies [35,46] included details on privacy, security, and ethical considerations, despite these factors being critical for user autonomy, consent, and overall intervention success, particularly for people with dementia. This limited attention reveals an important gap in the literature, underscoring the need for more robust ethical frameworks when designing, implementing, and evaluating such interventions.

The results demonstrate significant cost-effectiveness in adapting commonly available devices for dementia care and providing digital literacy training to support their use, as the technologies discussed in the 24 included studies generally cost approximately ≤US $3000. This cost is notably affordable compared to other types of interventions. For context, the NICE [74] classifies an intervention as cost-effective if it delivers one additional quality-adjusted life year, equivalent to one year of perfect health, at a cost between £20,000 (US $26,880) and £30,000 (US $40,320). In comparison, Carehome (2025) reports the average annual cost for standard residential care as £65,832 (approximately US $88,478), whereas specialized dementia nursing care escalates costs to an average of £80,808 (approximately US $108,606) annually, representing an additional £14,976 (approximately US $20,128) attributed directly to dementia-related needs [75]. Furthermore, pharmaceutical interventions such as aducanumab, intended to extend quality-adjusted life years, incur significantly higher annual costs, ranging from US $28,000 to US $56,000 (based on publicly reported estimates in media sources such as *Forbes* [76]). In addition, in 2015, the average annual cost of social dementia care in the United Kingdom was £15,060 (approximately US $20,241), with unpaid care cost at £14,620 (approximately US $19,649); by 2040, these costs are projected by the UK Care and Policy Evaluation Centre to rise significantly to £28,970 (approximately US $38,936), an increase of 92%, and £22,270 (approximately US $29,931), an increase of 52%, respectively [77]. Thus, at a cost of approximately ≤US $3000, the technological solutions discussed in the included studies clearly present substantial value, representing a highly cost-effective addition with broad accessibility.

Family caregivers play a pivotal role in managing the health and care of patients with dementia, as widely reported [78]. It is reasonable to assume that among two users of the same technology intervention, the one receiving extensive informal caregiver support is likelier to succeed. However, most of the included studies (15/24, 63%) did not measure or control for the management of caregiver support, which may have significantly influenced outcomes, particularly in studies that were not large RCTs.

In numerous studies (4/24, 17%), mobile-based user prompting and engagement strategies were used to maintain user involvement in the digital interventions, thereby enhancing accessibility. Features such as user-driven digital communication, reminders, gamification, and journaling positively impacted health outcomes, as also reported in other literature [79].

The efficacy of technology is often most perceptible in its absence rather than its presence, akin to how one may overlook one’s own nose despite its constant visibility. This inherent invisibility of smoothly functioning technology can render user evaluations challenging. Users may not actively recognize or appreciate technology that operates seamlessly and unobtrusively. Consequently, soliciting feedback on the performance of technology might yield more insightful information when framed in terms of operational effectiveness or in instances where technological failures are evident. Thus, we focused on extracting validated measures, aligned outcomes, and specific aspects of functionality known to be effective, as well as identifying points of technological breakdown that may provide a more accurate gauge of the impact and utility of technology in this study.

### Study Design Quality

This review included several excellent qualitative studies (10/24, 42%) of digital interventions, which provided valuable insights into the use and accessibility of self-management interventions. The quantitative studies (10/24, 42%) were also of good quality, offering complementary findings that helped isolate and quantify measured outcomes.

The absence of blinded RCTs among the included studies may introduce potential bias. However, this methodological limitation is justifiable, given that the primary aim was to identify and evaluate technologies actively used by participants as practical tools. Unlike pharmaceutical trials, it is often impractical or unfeasible to create placebo equivalents for interactive, technology-based interventions. Moreover, given the complexity of such interventions, traditional RCT designs may not adequately capture their multifaceted nature or the contextual factors influencing their effectiveness. In such cases, applying the Medical Research Council framework, which provides structured guidance for evaluating complex interventions, can help reduce bias and improve the reliability of findings.

In the context of technology tools, assessing real-world use metrics can offer valuable insights into their effectiveness. Important measures include adoption rates among the intended user group, user retention rates, total duration and frequency of use, and health care professionals’ willingness to invest in these tools, indicating potential for sustained use and impact.

### Limitations

This review is constrained by pronounced heterogeneity in study designs, intervention types, and outcome measures, rendering the calculation of pooled effect sizes or a meta‑analysis impracticable. Across the 24 included studies, we identified 135 distinct outcomes, each typically rated on a 5‑point Likert‑type scale and assessed at several time points. Applying a single framework such as the NOC would therefore have required appraising >16,000 data points and would have depended on subjective mapping decisions. We consequently adopted a structured narrative synthesis to show where measurable outcomes align with care targets and current guidance.

Our analysis relies chiefly on the NICE recommendations and selected NOC indicators. Although these frameworks are widely used, alternative benchmarks, particularly the ICHOM patient‑centered outcome set, were not examined in depth, which introduces potential regional and organizational bias. The evidence base is further weakened by the absence of blinded RCTs and the exclusion of non‑English publications, both of which may amplify selection and language bias. Collectively, these constraints highlight the need for standardized outcome sets, internationally harmonized guidelines, and rigorously designed trials in the evaluation of dementia care technologies.

### Conclusions

We conducted a systematic review of self-management technologies, including wearables, smart home technologies, and mobile apps, aimed at enhancing QoL and reducing dementia-associated behaviors in individuals with dementia. The results showed that self-management technologies demonstrate promising potential to enhance patient QoL and mitigate dementia-associated behaviors, although definitive conclusions regarding effectiveness are limited by the heterogeneity in study designs and outcomes. Strategies from the included studies encompassed machine-based guiding or prompting, cognitive support mechanisms, monitoring for abnormalities, sensory interventions, and the use of socially assistive robots.

The reviewed studies indicate that mobile apps have excellent potential for dementia care due to their adaptability to various desired outcomes identified through the NOC mapping (Figure 5). This flexibility is crucial for personalizing and adapting care for long-term conditions. Studies focusing on standardized care outcomes, typically those involving mobile apps, demonstrated greater potential for reaching a broader patient population. They also supported individualized care journeys over extended periods, accommodating necessary changes.

There was a lack of studies on atypical dementia types beyond Alzheimer disease; minoritized groups; self-care without caregiver support; and outcomes such as symptom status, self-control, risk control, safety, advanced needs (eg, care planning), and awareness guidance. To improve access to beneficial technologies and enhance digital literacy among patients with dementia, it is essential to address the digital divide, especially given the high configuration requirements of many technologies in the included studies. Providing educational and training opportunities can mitigate accessibility issues, making these essential tools more usable and accessible for those who would benefit the most.

The effectiveness of most technologies increases with greater involvement of individuals supporting the person with dementia, highlighting their potential in extensive care environments. Notably, these technologies demonstrate considerable utility in larger care settings where carers support self-management.

Limited standardization, interoperability issues between devices and software, and difficulties establishing clear clinical efficacy discourage collaborative efforts and the sustained refinement of a singular dementia care technology. Future studies should use a consistent battery of scientifically validated outcome assessments to reduce bias and facilitate future research improvements. Policy makers should subsidize digital literacy and accessibility for dementia care devices, promoting standards compliance for interoperability, with benefits measured against standardized outcomes such as those identified through the NOC mapping. Future studies could enhance result quality by using a standard control assessment to measure and manage modifiable disease factors in patients with dementia, such as hearing loss, physical activity, and social engagement, that could change during the study period.

## Appendix

Multimedia Appendix 1 Search strategy breakdown.

Multimedia Appendix 2 Study screening and exclusion criteria.

Multimedia Appendix 3 Reviewer agreement analysis (Cohen κ) .

Multimedia Appendix 4 Comparative feature analysis using the Pugh matrix.

Multimedia Appendix 5 National Institute for Health and Care Excellence quality standard 184 subgroup analysis.

Multimedia Appendix 6 Nursing Outcomes Classification subgroup analysis.

Multimedia Appendix 7 Mixed Methods Appraisal Tool ratings.

Multimedia Appendix 8 Critical Appraisal Skills Program traffic light checklist.

Multimedia Appendix 9 PRISMA checklist.

## Abbreviations

CASP: Critical Appraisal Skills Program
MMAT: Mixed Methods Appraisal Tool
NICE: National Institute for Health and Care Excellence
NOC: Nursing Outcomes Classification
PICO: population, intervention, comparison, and outcomes
PRISMA: Preferred Reporting Items for Systematic Reviews and Meta-Analyses
QoL: quality of life
QS: quality standard
RCT: randomized controlled trial
SNOMED-CT: Systemized Nomenclature of Medicine–Clinical Terms
