# Supplementary material for: Wearable Technology, Smart Home Systems, and Mobile Apps for the Self‑Management of Patient Outcomes in Dementia Care: Systematic Review
Source: J Med Internet Res. 2025 Aug 21;27:e65385. doi: 10.2196/65385 (PMC12411798; doi:10.2196/65385)
Supplement: Multimedia Appendix 1 [file jmir_v27i1e65385_app1.docx]

*Appendix 1. Search strategy breakdown (ran on 1/10/2023):*

| **PI~~C~~Ot** | **Description** | **Search string (Pubmed search ran on** **1/10/2023 below, other variations follow table)** | **Pubmed** | **Scopus** | **ACM Digital** | **EMBESCO: CINAHL** | **EMBESCO: PsycInfo** | **Web of Science** | **IEEE** | **Ovid Embase** | **Ovid Medline** |
| --- | --- | --- | --- | --- | --- | --- | --- | --- | --- | --- | --- |
| Patient | Patient cohort with most common forms of dementia (Alzheimer's disease ~60-80%, Vascular dementia ~10-20%, Lewy body dementia ~5-10% and Frontotemporal dementia ~5% ). | (("Dementia"[Mesh] OR "Alzheimer Disease"[Mesh]) OR (("dementi*"[tiab] OR "alzheimer*"[tiab]) OR ("Alzheimer* disease" OR "vascular dementia" OR "frontotemporal dementia" OR "Lewy body"))) AND | 168,354 | 64,156 | 249 | 36,236 | 28,577 | 23,470 | 1,192 |  |  |
| Intervention | Digital devices, and technologies commonly used with an aim to support individuals with dementia and improve their overall safety and quality of life. Focussed on established feature rich technology common platforms like smartwatch rather than specific vendor products like iWatch. | (((MH "Wearable Electronic Devices" OR MH "Mobile Applications" ) OR ( TI "technology" OR TI "smart*" OR AB "smart*" OR TI "wearable" OR AB "wearable" OR TI "mobile" OR AB "mobile" OR TI "mhealth" OR AB "mhealth")) AND ("smartwatch" OR "wearable device" OR "wearable technology" OR "smart home" OR "smart devices" OR "ambient assisted living" OR "cognitive assistance" OR "ambient intelligence" OR "internet of things" OR "telecare" OR "telehealth" OR "health technology" OR "digital health" OR "ehealth" OR "mobile health" OR "mhealth" OR "health apps" OR "mobile applications" OR "voice-activated" OR "multimodal" OR "health sensor" OR "gerontechnology" OR "domotics")) AND | 805,643 | 111,670 | 1,942 | 21,972 | 45,200 | 5,942 | 16,695 |  |  |
| Outcome | Patient-centred benefits that contribute towards alleviating cognitive or behavioural dementia symptoms or enhancing the quality of life for those with dementia (similar terms are aligned with Nursing Outcomes Classification (NOC)). | ((MH "Activities of Daily Living" OR MH "Quality of Life" OR MH "Independent Living" ) OR ("quality of life" OR "life satisfaction" OR "person-centered care" OR "personalized care" OR "individualized care" OR "tailored care" OR "engagement" OR "behavior control" OR "reduced depression" OR "reduced anxiety" OR "improved mood" OR "emotional control" OR "memory support" OR "memory enhancement" OR "stimulation" OR "improved cognition" OR "sense of purpose" OR "enhanced communication" OR "activities of daily living" OR "independent living" OR "slowed disease" OR "care plan" OR "care pathway" OR "rehabilitation" OR "pain management" OR "fall prevention" OR "decision-making" OR "*ed ability" OR "self-*")) | 774,473 | 497,626 | 37,837 | 208,739 | 140,421 | 77,215 | 131,235 |  |  |
| Time range |  | ((2013:2023[dp]) AND (English[la])) | - | - | - | - | - | - | - | - | - |
| Combined |  |  | 302 | 211 | 20 | 153 | 85 | 269 | 161 | 7 | 34 |

*Scopus Search (ran on 1/10/2023) :*

TITLE-ABS-KEY (

(

("Alzheimer* disease" OR "vascular dementia" OR "frontotemporal dementia" OR "Lewy body")

)

AND

(

("technology" OR "smart*" OR "wearable" OR "mobile" OR "mhealth")

AND

(

"smartwatch" OR "wearable device" OR "wearable technology" OR "smart home" OR

"smart devices" OR "ambient assisted living" OR "cognitive assistance" OR

"ambient intelligence" OR "internet of things" OR "telecare" OR "telehealth" OR

"health technology" OR "digital health" OR "ehealth" OR "mobile health" OR

"mhealth" OR "health apps" OR "mobile applications" OR "voice-activated" OR

"multimodal" OR "health sensor" OR "gerontechnology" OR "domotics"

)

)

AND

(

"quality of life" OR "life satisfaction" OR "person-centered care" OR

"personalized care" OR "individualized care" OR "tailored care" OR

"engagement" OR "behavior control" OR "reduced depression" OR "reduced anxiety" OR

"improved mood" OR "emotional control" OR "memory support" OR "memory enhancement" OR

"stimulation" OR "improved cognition" OR "sense of purpose" OR "enhanced communication" OR

"activities of daily living" OR "independent living" OR "slowed disease" OR

"care plan" OR "care pathway" OR "rehabilitation" OR "pain management" OR

"fall prevention" OR "decision-making" OR "*ed ability" OR "self-*"

)

)

*ACM Search (ran on 1/10/2023):*

Advanced mode

Abstract: (

("dementi*" OR "alzheimer*")

AND

("technology" OR "smart*" OR "wearable" OR "mobile" OR "mhealth")

)

Anywhere: (

(

(

"Alzheimer* disease" OR "vascular dementia" OR "frontotemporal dementia" OR "Lewy body"

)

)

AND

(

"cognitive assistance" OR "smartwatch" OR "wearable device" OR

"wearable technology" OR "smart home" OR "smart devices" OR

"ambient assisted living" OR "ambient intelligence" OR "voice-activated" OR

"internet of things" OR "iot" OR "mobile applications" OR

"mobile health" OR "ehealth" OR "mhealth" OR

"m-health" OR "health apps" OR "health technology" OR

"digital health" OR "telecare" OR "telehealth" OR

"multimodal" OR "adaptive" OR “health sensor" OR

"biometric sensor" OR "biosensor"

)

AND

(

"quality of life" OR "life satisfaction" OR "person-centered care" OR

"personalized care" OR "individualized care" OR "tailored care" OR

"engagement" OR "behavior control" OR "reduced depression" OR

"reduced anxiety" OR "improved mood" OR "emotional control" OR

"memory support" OR "memory enhancement" OR "stimulation" OR

"improved cognition" OR "sense of purpose" OR "enhanced communication" OR

"activities of daily living" OR "independent living" OR "slowed disease" OR

"care plan" OR "care pathway" OR "rehabilitation" OR

"pain management" OR "fall prevention" OR "decision-making" OR

"*ed ability" OR "self-*"

)

)

AND [E-Publication Date: (01/01/2013 TO 31/12/2023)]

*CINAHL Search (ran on 1/10/2023):*

(

(

MH "Dementia" OR MH "Alzheimer Disease"

) OR

(

(

TI "dementi*" OR AB "dementi*" OR TI "alzheimer*" OR AB "alzheimer*"

) OR

(

"Alzheimer* disease" OR "vascular dementia" OR "frontotemporal dementia" OR

"Lewy body"

)

)

) AND

(

(

(

MH "Wearable Electronic Devices" OR MH "Mobile Applications"

) OR

(

TI "technology" OR TI "smart*" OR AB "smart*" OR

TI "wearable" OR AB "wearable" OR TI "mobile" OR

AB "mobile" OR TI "mhealth" OR AB "mhealth"

)

) AND

(

"smartwatch" OR "wearable device" OR "wearable technology" OR

"smart home" OR "smart devices" OR "ambient assisted living" OR

"cognitive assistance" OR "ambient intelligence" OR "internet of things" OR

"telecare" OR "telehealth" OR "health technology" OR

"digital health" OR "ehealth" OR "mobile health" OR

"mhealth" OR "health apps" OR "mobile applications" OR

"voice-activated" OR "multimodal" OR "health sensor" OR

"gerontechnology" OR "domotics"

)

) AND

(

(

MH "Activities of Daily Living" OR MH "Quality of Life" OR MH "Independent Living"

) OR

(

"quality of life" OR "life satisfaction" OR "person-centered care" OR

"personalized care" OR "individualized care" OR "tailored care" OR

"engagement" OR "behavior control" OR "reduced depression" OR

"reduced anxiety" OR "improved mood" OR "emotional control" OR

"memory support" OR "memory enhancement" OR "stimulation" OR

"improved cognition" OR "sense of purpose" OR "enhanced communication" OR

"activities of daily living" OR "independent living" OR "slowed disease" OR

"care plan" OR "care pathway" OR "rehabilitation" OR "pain management" OR

"fall prevention" OR "decision-making" OR "*ed ability" OR "self-*"

)

)

*PsycInfo Search (ran on 1/10/2023):*

(

(

MH "Dementia" OR MH "Alzheimer Disease"

) OR

(

(

TI "dementi*" OR AB "dementi*" OR TI "alzheimer*" OR AB "alzheimer*"

) OR

(

"Alzheimer* disease" OR "vascular dementia" OR "frontotemporal dementia" OR

"Lewy body"

)

)

) AND

(

(

(

MH "Wearable Electronic Devices" OR MH "Mobile Applications"

) OR

(

TI "technology" OR TI "smart*" OR AB "smart*" OR

TI "wearable" OR AB "wearable" OR TI "mobile" OR

AB "mobile" OR TI "mhealth" OR AB "mhealth"

)

) AND

(

"smartwatch" OR "wearable device" OR "wearable technology" OR

"smart home" OR "smart devices" OR "ambient assisted living" OR

"cognitive assistance" OR "ambient intelligence" OR "internet of things" OR

"telecare" OR "telehealth" OR "health technology" OR "digital health" OR

"ehealth" OR "mobile health" OR "mhealth" OR "health apps" OR "mobile applications" OR

"voice-activated" OR "multimodal" OR "health sensor" OR "gerontechnology" OR "domotics"

)

) AND

(

(

MH "Activities of Daily Living" OR MH "Quality of Life" OR MH "Independent Living"

) OR

(

"quality of life" OR "life satisfaction" OR "person-centered care" OR

"personalized care" OR "individualized care" OR "tailored care" OR

"engagement" OR "behavior control" OR "reduced depression" OR

"reduced anxiety" OR "improved mood" OR "emotional control" OR

"memory support" OR "memory enhancement" OR "stimulation" OR

"improved cognition" OR "sense of purpose" OR "enhanced communication" OR

"activities of daily living" OR "independent living" OR "slowed disease" OR

"care plan" OR "care pathway" OR rehabilitation" OR "pain management" OR

"fall prevention" OR "decision-making" OR "*ed ability" OR "self-*"

)

)

*Web of Science Search (ran on 1/10/2023):*

(

(

(

TS="Dementia" OR TS="Alzheimer Disease"

)

) OR

(

(

AB="dementi*" OR AB="alzheimer*"

) OR

ALL=(

"Alzheimer* disease" OR "vascular dementia" OR "frontotemporal dementia" OR

"Lewy body"

)

)

) AND

(

(

(

TS="Wearable Electronic Devices" OR TS="Mobile Applications"

) OR

(

TI="technology" OR AB="smart*" OR AB="wearable" OR

AB="mobile" OR AB="mhealth"

)

) AND

ALL=(

"smartwatch" OR "wearable device" OR "wearable technology" OR

"smart home" OR "smart devices" OR "ambient assisted living" OR

"cognitive assistance" OR "ambient intelligence" OR

"internet of things" OR "telecare" OR "telehealth" OR

"health technology" OR "digital health" OR "ehealth" OR

"mobile health" OR "mhealth" OR "health apps" OR

"mobile applications" OR "voice-activated" OR "multimodal" OR

"health sensor" OR "gerontechnology" OR "domotics"

)

) AND

(

(

TS="Activities of Daily Living" OR TS="Quality of Life" OR TS="Independent Living"

) OR

ALL=(

"quality of life" OR "life satisfaction" OR "person-centered care" OR

"personalized care" OR "individualized care" OR "tailored care" OR

"engagement" OR "behavior control" OR "reduced depression" OR

"reduced anxiety" OR "improved mood" OR "emotional control" OR

"memory support" OR "memory enhancement" OR "stimulation" OR

"improved cognition" OR "sense of purpose" OR "enhanced communication" OR

"activities of daily living" OR "independent living" OR "slowed disease" OR

"care plan" OR "care pathway" OR "rehabilitation" OR "pain management" OR

"fall prevention" OR "decision-making" OR "improved ability" OR

"self-care"

)

)

*IEEE Search (ran on 1/10/2023):*

(

(

(

"Mesh_Terms":"Dementia" OR "Mesh_Terms":"Alzheimer Disease"

)

) OR

(

(

"Abstract":"dementi*" OR "Abstract":"alzheimer*"

) OR

(

"Full Text Only":"Alzheimer* disease" OR "Full Text Only":"vascular dementia" OR

"Full Text Only":"frontotemporal dementia" OR "Full Text Only":"Lewy body"

)

)

) AND

(

(

(

"Mesh_Terms":"Wearable Electronic Devices" OR "Mesh_Terms":"Mobile Applications"

) OR

(

"Document Title":"technology" OR "Abstract":"smart*" OR "Abstract":"wearable" OR

"Abstract":"mobile" OR "Abstract":"mhealth"

)

) AND

(

"Full Text Only":"smartwatch" OR "Full Text Only":"wearable device" OR

"Full Text Only":"wearable technology" OR "Full Text Only":"wearable technology" OR

"Full Text Only":"smart home" OR "Full Text Only":"smart devices" OR

"Full Text Only":"ambient assisted living" OR "Full Text Only":"cognitive assistance" OR

"Full Text Only":"ambient intelligence" OR "Full Text Only":"internet of things" OR

"Full Text Only":"telecare" OR "Full Text Only":"telehealth" OR

"Full Text Only":"health technology" OR "Full Text Only":"digital health" OR

"Full Text Only":"ehealth" OR "Full Text Only":"mobile health" OR

"Full Text Only":"mhealth" OR "Full Text Only":"health apps" OR

"Full Text Only":"mobile applications" OR "Full Text Only":"voice-activated" OR

"Full Text Only":"multimodal" OR "Full Text Only":"health sensor" OR

"Full Text Only":"gerontechnology" OR "Full Text Only":"domotics"

)

) AND

(

(

"Mesh_Terms":"Activities of Daily Living" OR "Mesh_Terms":"Quality of Life" OR

"Mesh_Terms":"Independent Living"

) OR

(

"Full Text Only":"quality of life" OR "Full Text Only":"life satisfaction" OR

"Full Text Only":"person-centered care" OR "Full Text Only":"personalized care" OR

"Full Text Only":"individualized care" OR "Full Text Only":"tailored care" OR

"Full Text Only":"engagement" OR "Full Text Only":"behavior control" OR

"Full Text Only":"reduced depression" OR "Full Text Only":"reduced anxiety" OR

"Full Text Only":"improved mood" OR "Full Text Only":"emotion control" OR

"Full Text Only":"memory support" OR "Full Text Only":"memory enhancement" OR

"Full Text Only":"stimulation" OR "Full Text Only":"improved cognition" OR

"Full Text Only":"sense of purpose" OR "Full Text Only":"enhanced communication" OR

"Full Text Only":"activity of daily living" OR "Full Text Only":"independent living" OR

"Full Text Only":"slowed disease" OR "Full Text Only":"care plan" OR

"Full Text Only":"care pathway" OR "Full Text Only":"rehabilitation" OR

"Full Text Only":"pain management" OR "Full Text Only":"fall prevention" OR

"Full Text Only":"decision-making" OR "Full Text Only":"improved ability" OR

"Full Text Only":"self-care"

)

)

*Ovid Embase Search (ran on 1/10/2023):*

(

(

kf="Dementia" OR kf="Alzheimer Disease"

) OR

(

(

ab="dementi*" OR ab="alzheimer*"

) OR

(

"Alzheimer* disease" OR "vascular dementia" OR "frontotemporal dementia" OR "Lewy body"

)

)

) AND

(

(

(

"Wearable Electronic Devices" OR "Mobile Applications"

) OR

(

ti="technology" OR ab="smart*" OR ab="wearable" OR ab="mobile" OR ab="mhealth"

)

) AND

(

"smartwatch" OR "wearable device" OR "wearable technology" OR

"smart home" OR "smart devices" OR "ambient assisted living" OR

"cognitive assistance" OR "ambient intelligence" OR "internet of things" OR

"telecare" OR "telehealth" OR "health technology" OR

"digital health" OR "ehealth" OR "mobile health" OR

"mhealth" OR "health apps" OR "mobile applications" OR

"voice-activated" OR "multimodal" OR "health sensor" OR

"gerontechnology" OR "domotics"

)

) AND

(

(

kf="Activities of Daily Living" OR kf="Quality of Life" OR

kf="Independent Living"

) OR

(

"quality of life" OR "life satisfaction" OR "person-centered care" OR

"personalized care" OR "individualized care" OR "tailored care" OR

"engagement" OR "behavior control" OR "reduced depression" OR

"reduced anxiety" OR "improved mood" OR "emotional control" OR

"memory support" OR "memory enhancement" OR "stimulation" OR

"improved cognition" OR "sense of purpose" OR "enhanced communication" OR

"activities of daily living" OR "independent living" OR

"slowed disease" OR "care plan" OR "care pathway" OR

"rehabilitation" OR "pain management" OR "fall prevention" OR

"decision-making" OR "improved ability" OR "self-care"

)

) AND

(

(2013:2023[dp]) AND (English[la])

)

*Ovid medline (ran on 1/10/2023):*

(

(

kf="Dementia" OR kf="Alzheimer Disease"

) OR

(

(

ab="dementi*" OR ab="alzheimer*"

) OR

(

"Alzheimer* disease" OR "vascular dementia" OR "frontotemporal dementia" OR "Lewy body"

)

)

) AND

(

(

(

"Wearable Electronic Devices" OR "Mobile Applications"

) OR

(

ti="technology" OR ab="smart*" OR ab="wearable" OR ab="mobile" OR ab="mhealth"

)

) AND

(

"smartwatch" OR "wearable device" OR "wearable technology" OR

"smart home" OR "smart devices" OR "ambient assisted living" OR

"cognitive assistance" OR "ambient intelligence" OR "internet of things" OR

"telecare" OR "telehealth" OR "health technology" OR

"digital health" OR "ehealth" OR "mobile health" OR

"mhealth" OR "health apps" OR "mobile applications" OR

"voice-activated" OR "multimodal" OR "health sensor" OR

"gerontechnology" OR "domotics"

)

) AND

(

(

kf="Activities of Daily Living" OR kf="Quality of Life" OR kf="Independent Living"

) OR

(

"quality of life" OR "life satisfaction" OR "person-centered care" OR

"personalized care" OR "individualized care" OR "tailored care" OR

"engagement" OR "behavior control" OR "reduced depression" OR

"reduced anxiety" OR "improved mood" OR "emotional control" OR

"memory support" OR "memory enhancement" OR "stimulation" OR

"improved cognition" OR "sense of purpose" OR "enhanced communication" OR

"activities of daily living" OR "independent living" OR "slowed disease" OR

"care plan" OR "care pathway" OR "rehabilitation" OR

"pain management" OR "fall prevention" OR "decision-making" OR

"improved ability" OR "self-care"

)

) AND

(

(2013:2023[dp]) AND (English[la])

)
