# Supplementary material for: Wearable Technology, Smart Home Systems, and Mobile Apps for the Self‑Management of Patient Outcomes in Dementia Care: Systematic Review
Source: J Med Internet Res. 2025 Aug 21;27:e65385. doi: 10.2196/65385 (PMC12411798; doi:10.2196/65385)
Supplement: Multimedia Appendix 2 [file jmir_v27i1e65385_app2.docx]

#### Appendix 2. Study screening and exclusion criteria.

| Priority | Criterion | Assertion/test | Exclusion reason | Diagnostic annotation |
| --- | --- | --- | --- | --- |
| 1 | Does the academic paper cover adults with any of these conditions: Alzheimer's Disease, Vascular Dementia, Frontotemporal Dementia, Dementia with Lewy Bodies, or Mixed Dementia? | If not true | Wrong population | The study focuses on **adults with any of the five major types of dementia**. Other forms of dementia are included only if they are not explicitly categorized, and their results are directly applicable to these major types. ***Individuals without dementia, informal carers, or health professionals, those with Mild Cognitive Impairment (MCI), and those with rarer dementia forms, especially where symptoms, progression, and treatment differ markedly, are excluded*** from this study. |
| 2 | Does the academic paper evaluate the **impact on quality of life and behaviours related to dementia** in study participants with dementia after they have undergone a digital intervention? | If not true | Wrong outcome | Quality of life (QOL) is defined by WHO as "an individual's perception of their position in life in the context of the culture and value systems in which they live and in relation to their goals, expectations, standards and concerns". Dementia-associated behaviours are symptoms affecting mood, perception, and conduct, often seen in dementia patients. Key examples include agitation, aggression, apathy, confusion, delusions, emotional dysregulation/mood swings, hallucinations, repetition, sleep disturbances, and wandering. The user's behaviour may also be mentioned in studies related to the interaction with the technology itself, but this is not relevant to this inclusion rule! |
| 3 | Is there a method described in the academic paper for assessing **changes in quality of life** or **alterations in behaviours associated with dementia** in the study participants who have dementia? | If not true | Wrong measurement | The study must have some method to measure changes in quality of life or dementia-associated behaviour changes for people in the study with dementia. *Studies that solely explore acceptability, feasibility, or technology without providing quantifiable results should be excluded. Exclude studies with insignificant, subjective, or non-repeatable outcome change measurements*. Study outcomes ideally should be determined using repeatable, scientifically validated tests or assessments ( for QoL: QoL-AD, ADRQL, DEMQOL, EQ-5D, EQ-5D-3L, MQOL, Q-LES-Q, QOLS, WHOQOL-OLD, WHOQOL-BREF, ICECAP-O, HRQOL, AQoL-8D, PWI, SF-36, geriatric quality of life scale) (for behaviours: RMBPC, BEHAVE-AD, DSQIID, Cognistat, NPI-Q, RBMT, HDS, MENFIS, The Brief COPE). Also acceptable are objective measurements such as are made through RCTs, with controls, using standardised benchmarks or with patient-centred measurements such as PROMs or PREMs. |
| 4 | Does the academic paper evaluate an intervention that could serve as a self-management tool for individuals with dementia? | If not true | Wrong study type | The study types should be **self-management intervention technologies designed for use by people with dementia**. This includes tools like prompters, reminders, guides, virtual assistants, and decision-support systems. It *excludes technologies applied to dementia patients without their active involvement, such as observational monitoring, tracking, surveillance, and passive devices concealed from them*. Also ***excluded are technologies solely for health professionals, like those used in lab work, blood tests, medical imaging (PET, CT scans), and APPs for professionals or carers***. Only technologies that offer self-management capabilities for individuals with dementia are relevant. |
| 5 | Is the academic paper a firsthand, scientific report detailing the outcomes of a technological intervention, presented in a peer-reviewed article or conference paper? It should not be a systematic review, scoping review, commentary, general review, or an editorial article. | If not true | Wrong study design | The study design should be a peer-reviewed article or conference paper that specifically details a technology intervention and its measured outcomes. *Exclude systematic reviews, scoping reviews, commentaries, general reviews, editorials, and oral/video presentations*. |
| 6 | Is the academic paper published in English between 2013 and October 2023? | If not true | Out of scope | The study should be published in English between January 1, 2013, and September 30, 2023. |
