# Supplementary material for: Wearable Technology, Smart Home Systems, and Mobile Apps for the Self‑Management of Patient Outcomes in Dementia Care: Systematic Review
Source: J Med Internet Res. 2025 Aug 21;27:e65385. doi: 10.2196/65385 (PMC12411798; doi:10.2196/65385)
Supplement: Multimedia Appendix 3 [file jmir_v27i1e65385_app3.docx]

### Appendix 3. Reviewer agreement analysis (Cohen κ)

#### Review agreement for record screening

Each reviewer screened articles using the criteria in the above table, excluding those that met these criteria and marking uncertain ones as '?Maybe'. Of the 835 articles, reviewers unanimously agreed to exclude 720 and include 39. The remaining 76 articles were further evaluated in a review meeting, where a third reviewer was also present.

| WH\ GC | ?Maybe | Exclude |
| --- | --- | --- |
| ?Maybe | 39 | 62 |
| Exclude | 14 | 720 |

The Kappa score, a reliable statistical indicator of inter-rater agreement for qualitative assessments like this review, was calculated using Cohen's Kappa coefficient. With a score of 0.472, it signifies moderate agreement between the two reviewers, surpassing what would be expected by chance. The calculation details are provided below:

Observed Agreement (Po):


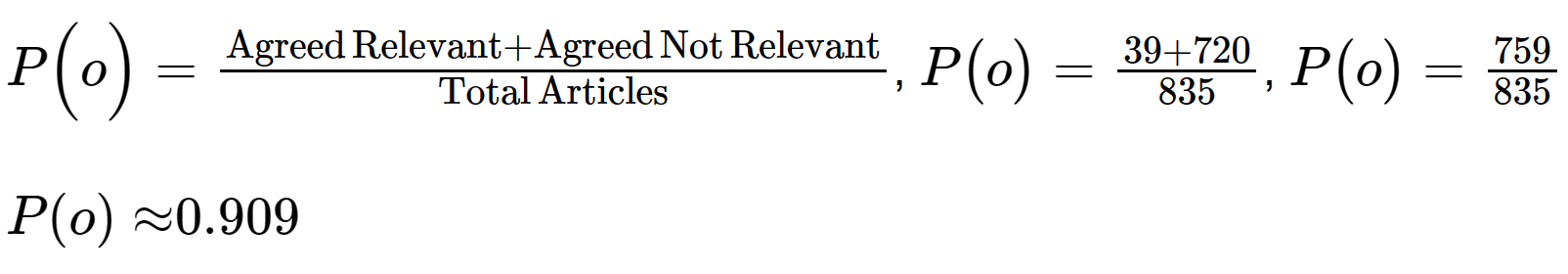


Expected Agreement by Chance (Pe):


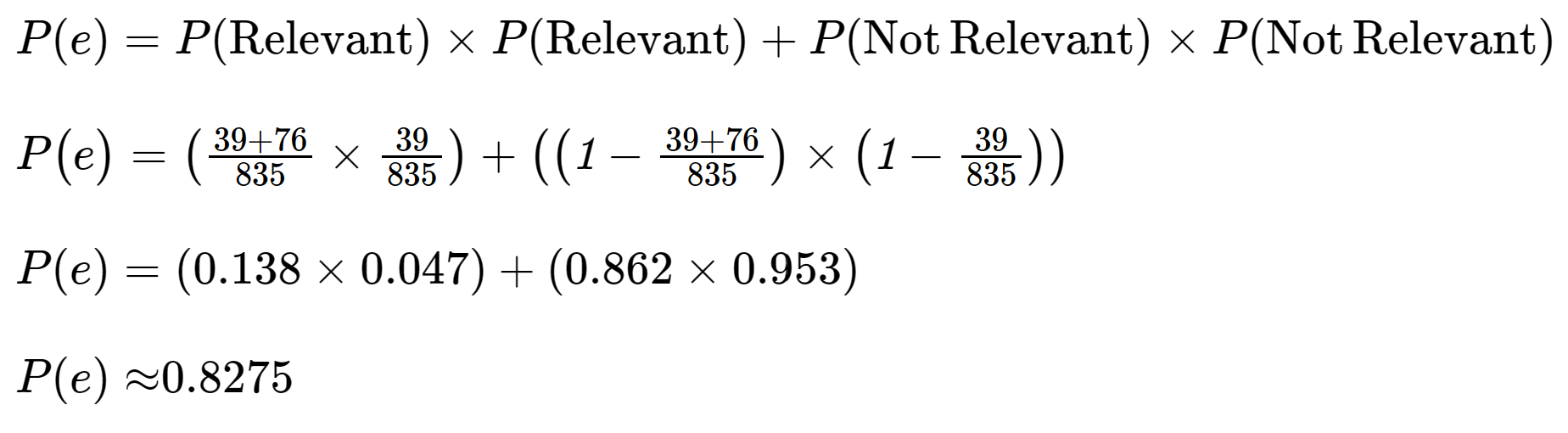


Kappa Score Calculation:


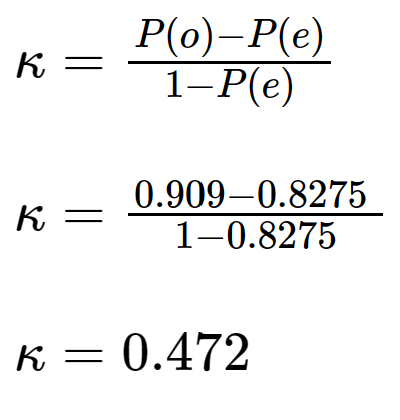


This is a moderate level of agreement achieved blind prior to discussion and perfect agreement.

#### Review agreement for full-text screening

The abstract review meeting addressed the 76 articles marked for exclusion by only one reviewer, discussing the reasons for potential exclusion. Notes were made on these articles, detailing the reasons, and re-evaluating them after clarifying the screening criteria. Key observations included that many articles mentioned dementia as a study focus but did not involve participants with dementia or mention the quality of life or behaviours in the introduction without assessing changes in these outcomes. Additionally, a few clerical errors were identified and corrected. After this second screening phase, 783 articles were unanimously excluded in complete agreement without any discrepancy in the assessments or ratings.

| WH \ GC | ?Maybe | Exclude |
| --- | --- | --- |
| ?Maybe | 52 | 0 |
| Exclude | 0 | 783 |

A meeting was organized to begin the full-text screen review of the remaining 52 articles that have been classified as '?Maybe' in the preceding process.

Full-text screening

The 52 articles full-text were reviewed independently and blindly by two reviewers. Each article was included or excluded for given reasons by a screener.

| WH \ GC | Include | Exclude |
| --- | --- | --- |
| Include | 22 | 5 |
| Exclude | 6 | 19 |

Observed Agreement (Po):


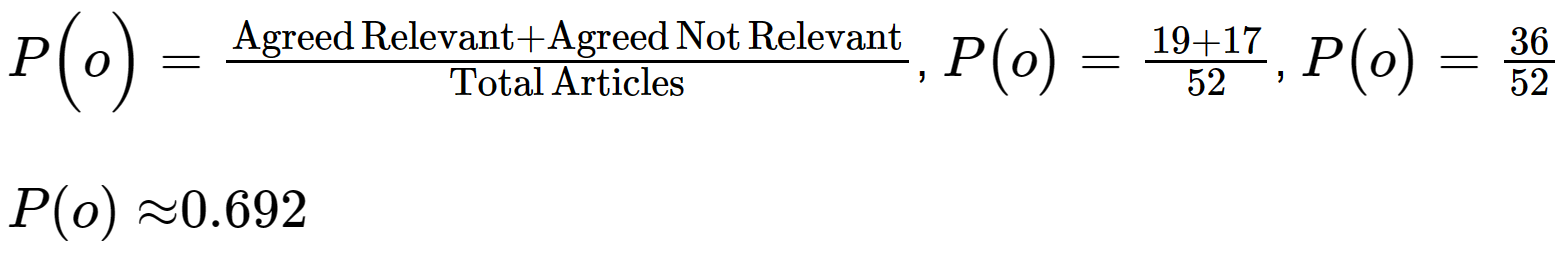


Expected Agreement by Chance (Pe):


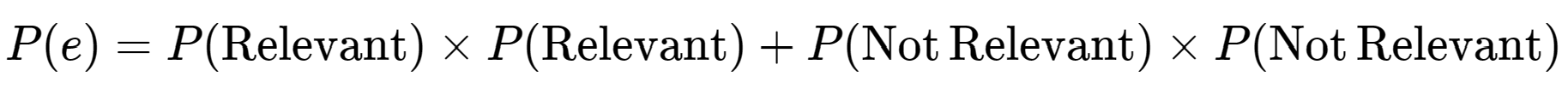


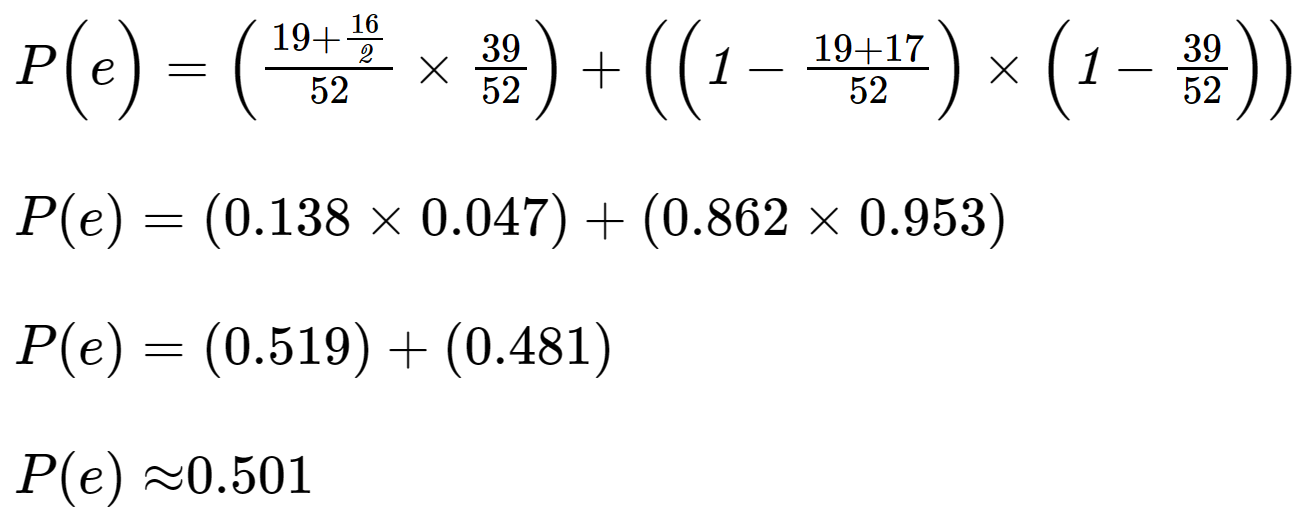


Kappa Score Calculation:


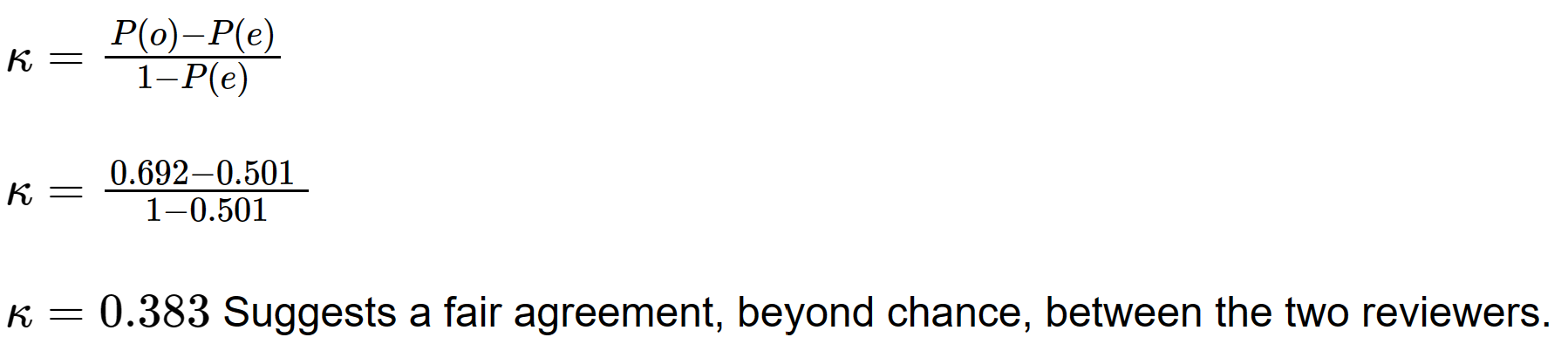


This is statistically a fair level of agreement achieved with the blind review prior to discussion and arriving at perfect agreement.
