# Supplementary material for: Wearable Technology, Smart Home Systems, and Mobile Apps for the Self‑Management of Patient Outcomes in Dementia Care: Systematic Review
Source: J Med Internet Res. 2025 Aug 21;27:e65385. doi: 10.2196/65385 (PMC12411798; doi:10.2196/65385)
Supplement: Multimedia Appendix 4 [file jmir_v27i1e65385_app4.docx]

###### Appendix 4. Comparative feature analysis using the Pugh matrix

| Article | | Duration | Participants | Disease | | | | | Environment | | | NOC | QoL measures | | Behavioural measures | | Total Score |
| --- | --- | --- | --- | --- | --- | --- | --- | --- | --- | --- | --- | --- | --- | --- | --- | --- | --- |
| [ref.] | Year, Authors | Days | No. | AD | VD | FtD | LBD | MD | Domi (1\|0) | Mobl (1\|0) | Clnc (1\|0) | Score | Measures | Score | Measures | Score | Score |
| Weighting, scoring | | x 0.05 | x 0.05 | - | - | - | - | - | x 5\|0 | x 5\|0 | - | x 0.2 | - | (0-5) | - | (0-5) | - |
| [1] | 2023, Rossetto, F., et al. | 42 | 21 | 21 | 0 | 0 | 0 | 0 | 1 | 1 | 1 | 15 | Montreal Cognitive Assessment (MoCA); Verbal Fluency Test (FAS and CAT); Trail Making Test (TMT Parts A and B); Free and Cued Selective Reminding Test (FCSRT); | 2 | None | 0 | 18.15 |
|  |  | Context: Use of ABILITY Digital Platform. Implementing the ABILITY digital-health home intervention to improve cognitive and behavioural abilities in individuals within the Alzheimer’s disease continuum. The intervention is based on the concept that digital platforms can enhance cognitive and behavioural abilities in people with Alzheimer’s disease. Moderate to large effect sizes on global cognition and language at 6 weeks and sustained at 12 months. Improved adherence (81% vs 62%). | | | | | | | | | |  |  |  |  |  |  |
| [2] | 2022, Menengiç, K.N., et al. | 42 | 10 | 10 | 0 | 0 | 0 | 0 | 1 | 1 | 1 | 21 | Mini-Mental State Examination; Timed Up and Go Test; 5 Times Sit and Stand Test; One-leg Stance Test; Geriatric Depression Scale-Short Form; Beck Anxiety Scale; | 3 | Katz Activities of Daily Living Scale; Functional Independence Measure; Geriatric Depression Scale-Short Form; Beck Anxiety Scale; Zarit Caregiver Burden Inventory; Warwick Edinburgh Well-being Scale; | 4 | 22.6 |
|  |  | Context: Motor-Cognitive Dual-Task Exercise via Telerehabilitation. Using telerehabilitation to deliver motor-cognitive dual-task exercise treatment for enhancing cognitive and physical functions in Alzheimer's disease. The intervention is based on the hypothesis that motor-cognitive dual-task exercise can improve cognitive and physical functions in AD patients. Statistically significant improvements in cognition (MMSE≈ +?) and mobility (TUG, 5×STS) but reported without effect sizes. | | | | | | | | | |  |  |  |  |  |  |
| [3] | 2020, Kerkhof, Y., et al. | 91 | 7 | 0 | 0 | 0 | 0 | 0 | 1 | 1 | 0 | 31 | DQoL(Dementia Quality of Life), EQ-5D-5L, EQ-VAS, TOPICS-MDS, Dutch version of the 10-item General Self-Efficacy Scale (D-GSE scale); Usability of FindMyApps Tool; Effectiveness of FindMyApps in Engagement; 12-item Experienced Autonomy List (EAL); | 5 | Dutch version of the 10-item General Self-Efficacy Scale (D-GSE scale); Dementia Quality of Life scale (DQoL); Self-Management Ability Scale (SMAS-30); Pleasant Activities List (PAL); Adult Social Care Outcomes Toolkit (ASCOT); 12-item Experienced Autonomy List (EAL); WHO Quality of Life-100 (WHOQOL-100); | 5 | 31.1 |
|  |  | Context: FindMyApps Training and Support. App Selection and Usage. The goal is to support individuals with mild dementia in managing their daily lives and engaging in meaningful activities, addressing the challenges they face due to cognitive impairments. Past evidence indicates that tablet-based interventions can be effective in supporting self-management and engagement in meaningful activities among people with mild dementia. Direction of effects generally favoured FindMyApps on several self-management sub scales and pleasant social activities (ηp² 0.12–0.42), but CIs unreported and p values non-significant; clinical importance uncertain. | | | | | | | | | |  |  |  |  |  |  |
| [4] | 2022, Dinesen, B., et al. | 84 | 42 | 20 | 0 | 0 | 0 | 0 | 1 | 1 | 1 | 6 | Participant Observations, semi structured Focus Group Interviews. | 1 | World Health Organization-5 (WHO-5) Well-being Index, Face Scale, semi structured Focus Group Interviews. | 3 | 21.5 |
|  |  | Context: Use of LOVOT in Dementia Care. Improving interaction, communication, and emotional well-being using the LOVOT robot. Past evidence indicates that social robots like PARO have improved mood, social engagement, and reduced negative emotions among persons with dementia. WHO 5: No clinically important change (≥10 point) in either cohort over time. – Face Scale: Median scores trended to more positive expressions post session, but no numerical effect sizes or CIs supplied. A few participants experienced emotional overstimulation requiring session termination. | | | | | | | | | |  |  |  |  |  |  |
| [5] | 2022, Han, S.S., K. White, and E. Cisek | 42 | 7 | 7 | 0 | 0 | 0 | 0 | 1 | 1 | 0 | 5 | Alzheimer’s Disease Cooperative Study - Activities of Daily Living Inventory (ADCS-ADL), Margaret Blenker Research Center (MBRC) Caregiver Strain instrument, Caregiver Net Promoter-Type Survey. | 1 | Alzheimer’s Disease Cooperative Study - Activities of Daily Living Inventory (ADCS-ADL), Margaret Blenker Research Center (MBRC) Caregiver Strain instrument, Quality of Life (QOL-18), Caregiver Net Promoter-Type Survey. | 3 | 17.45 |
|  |  | Context: Visual Mapping Assistive Technology. Improving daily living activities and reducing caregiver burden for individuals with Alzheimer’s disease and related dementias. Research focused on assessing the feasibility and effectiveness of the MapHabit System (MHS), a visual mapping software application, in supporting daily activities and reducing caregiver burden. Statistically significant within group gains in ADL (+2.6/53) and modest between group advantages for caregiver strain ( 0.64 on 0 3 scale) and QoL (+0.27/5). Clinical importance unclear; between group ADL effect non-significant. | | | | | | | | | |  |  |  |  |  |  |
| [6] | 2022, Larnyo, E., et al. | 1 | 262 | - | - | - | - | - | 1 | 1 | 0 | 11 | SF-36, Extended Unified Theory of Acceptance and Use of Technology (UTAUT), The Mini-Mental Status Examination (MMSE). | 2 | Quality of Life (QoL) Assessment Using SF-36, Extended Unified Theory of Acceptance and Use of Technology (UTAUT). | 2 | 28.35 |
|  |  | Context: Adoption and Use of Healthcare Wearable Devices. Using healthcare wearable devices to enhance the quality of life for people with dementia by monitoring health and facilitating timely interventions. Study based on the extended UTAUT model and SF-36 quality of life instrument to assess the impact of wearable device usage. | | | | | | | | | |  |  |  |  |  |  |
| [7] | 2022, Freytag, J., et al. | 122 | 19 | - | - | - | - | - | 1 | 0 | 0 | 11 | The World Health Organization Quality of Life measure (WHO-QOL-OLD), The Multimorbid Treatment Burden Questionnaire (MTBQ), Community Integration Questionnaire (CIQ), Life Space Function, Wearable Sensor Data Analysis, Self-Report Assessments of Mobility, Montreal Cognitive Assessment (MoCA). Katz Index of Independence in Activities of Daily Living (ADL), Lawton–Brody Instrumental Activities of Daily Living (IADL | 4 | The Multimorbid Treatment Burden Questionnaire (MTBQ); Life Space Function; Wearable Sensor Data Analysis; Self-Report Assessments of Mobility | 2 | 20.25 |
|  |  | Context: Integration of Wearable Sensor Data in Clinical Care. The goal is to align treatment with patients' self-determined healthcare priorities in dementia care, using wearable sensors to facilitate the measurement of goal achievement. Research focused on the feasibility of using wearable sensors to monitor healthcare goals, differences in function after goal-setting visits, and goal achievement in patients with dementia. Feasibility shown; some individuals increased physical activity and met self-stated goals. Clinical relevance uncertain because magnitude of change and relation to patient centred outcomes not quantified. | | | | | | | | | |  |  |  |  |  |  |
| [8] | 2021, Kelleher, J., et al. | 91 | 14 | - | - | - | - | - | 1 | 0 | 0 | 15 | MapHabit System Usability and Impact Assessment. | 1 | Repeatable Battery for the Assessment of Neuropsychological Status (RBANS), MapHabit System Usability and Impact Assessment. | 3 | 15.25 |
|  |  | Context: MapHabit Visual Mapping. Using the MapHabit app to aid individuals with cognitive impairment in recalling steps to independently complete ADLs, thereby improving functional ability and quality of life. The intervention is based on the concept that visual aids can help in recalling steps for ADLs, leveraging the procedural memory system. Statistically significant perceived improvements in some functional and psychosocial domains; magnitude unclear (no effect sizes/CIs). | | | | | | | | | |  |  |  |  |  |  |
| [9] | 2021, Howard, R., et al. | 728 | 248 | - | - | - | - | - | 0 | 0 | 0 | 7 | EQ-5D Quality of Life Assessment, BADLS (Bristol Activities of Daily Living Scale), Time to Institutionalization Assessment, Cost-Effectiveness Analysis | 4 | SMMSE (Standardised Mini-Mental State Examination), BADLS (Bristol Activities of Daily Living Scale), Cost-Effectiveness Analysis | 2 | 56.2 |
|  |  | Context: Assessment and Installation of ATT Devices. Goal aimed at assessing the effectiveness of assistive technology and telecare in supporting independent living in people with dementia. Research focused on evaluating whether ATT could enable people with dementia to maintain safe independent living for longer in their homes. The study compared full ATT intervention with limited control ATT. No statistically or clinically significant delay in institutionalisation; small but significant decrement in participant reported QoL. | | | | | | | | | |  |  |  |  |  |  |
| [10] | 2021, Harris, N., et al. | 28 | 14 | 7 | 4 | - | - | 4 | 0 | 0 | 0 | 9 | BADLS (Bristol Activities of Daily Living Scale), Tablet-Based Prompter Usability and Effectiveness Assessment, Alzheimer’s Disease Knowledge Scale (ADKS), Sense of Competence Questionnaire (SCQ), Log of Prompter Use, Goal Attainment Scale (GAS), Rating of Success. | 2 | ACE-III (Addenbrooke's Cognitive Examination-III), BADLS (Bristol Activities of Daily Living Scale), Tablet-Based Prompter Usability and Effectiveness Assessment, Log of Prompter Use, Goal Attainment Scale (GAS), California Verbal Learning Task (CVLT-II), Trail Making Component of the Delis Kaplan Executive Functions System Test Battery (D-KEFS-TM), Rating of Success. | 4 | 9.9 |
|  |  | Context: Tablet-Based Digital Prompting. Assisting individuals with mild to moderate dementia in completing multistep tasks to enhance their autonomy and quality of life. Research focused on evaluating the feasibility of a tablet-based prompter for individuals living with dementia. The study involved carers loading instructions for completing tasks onto the prompter, which the person with dementia then used independently. 73 % of dyads met at least two personalised goals; higher task use frequency correlated with goal attainment, suggesting potential usability and functional gain. However, without a control condition, the magnitude of benefit versus usual care is unknown. | | | | | | | | | |  |  |  |  |  |  |
| [11] | 2021, Goodall, G., et al. | 84 | 7 | 1 | 1 | - | 1 | - | 0 | 0 | 0 | 9 | SENSE-GARDEN Experience Evaluation. | 1 | SENSE-GARDEN Experience Evaluation. | 1 | 8.35 |
|  |  | Context: SENSE-GARDEN Intervention. Goal aimed at maintaining the sense of self in people with dementia, addressing the crisis of identity loss due to dementia. Research focused on how the SENSE-GARDEN intervention, using digital technologies and multisensory stimuli, can facilitate individualized, meaningful activities for people with dementia. Participants and caregivers perceived better communication, emotional expression and relationship quality during/after sessions. Magnitude cannot be quantified; benefits are psychosocial and context dependent. | | | | | | | | | |  |  |  |  |  |  |
| [12] | 2020, Gall, D., et al. | 183 | 8 | - | - | - | - | - | 0 | 0 | 0 | 13 | CareShare System Usability and Impact Assessment. | 1 | CareShare System Usability and Impact Assessment. | 1 | 14.15 |
|  |  | Context: Implementation of CareShare for Knowledge Management. The goal is to enhance the quality of person-centered care for individuals with dementia by reducing communication gaps through self-organizing knowledge management, using a mobile application. Research focused on evaluating the impact of a collaborative communication system, CareShare, on enhancing person-centred care in dementia. The study involved significant others of residents with severe dementia and professional caregivers using a mobile application. Reported improvements are qualitative, anecdotal and not measured on clinically meaningful scales. True effect size unknown. | | | | | | | | | |  |  |  |  |  |  |
| [13] | 2020, Ferry, F., et al. | 91 | 30 | - | - | - | - | - | 0 | 0 | 0 | 28 | DEMQOL and DEMQOL Proxy, Health-Related Quality of Life (HRQoL), Euroqol EQ-5D, Client Socio-demographic and Service Receipt Inventory (CSRI), | 5 | None | 0 | 17.65 |
|  |  | Context: InspireD App for Reminiscence. The goal is to improve the quality of life and wellbeing of persons living with dementia through a home-based, individual-specific reminiscence intervention, facilitated by an iPad app. Research focused on the impact of a reminiscence intervention using technology to support people with dementia. The study aimed to estimate costs and quality of life associated with the intervention and inform future cost-effectiveness analysis. Modest mean improvement in EQ 5D (+0.07) and DEMQOL over 3 months; clinical significance uncertain without variability data. | | | | | | | | | |  |  |  |  |  |  |
| [14] | 2020, Øksnebjerg, L., et al. | 91 | 19 | - | - | - | - | - | 0 | 0 | 0 | 116 | EQ-5D-5L, EQ-5D Visual Analog Scale (VAS), Individual Goal Attainment, Adoption of the ReACT App, Bangor Goal Setting Interview (BGSI), ICECAP-O | 3 | Addenbrooke's Cognitive Examination (ACE), Repeatable Battery for the Assessment of Neuropsychological Status (RBANS), Individual Goal Attainment, Adoption of the ReACT App, Bangor Goal Setting Interview (BGSI), Mini-Mental State Examination (MMSE) | 4 | 35.7 |
|  |  | Context: Adoption of the ReACT App. Implementing a combined approach of cognitive rehabilitation and self-management to enhance coping mechanisms and promote the adoption of assistive technology in early-stage dementia. The intervention is based on the need for individualized support for coping and self-management in dementia, with a focus on integrating cognitive rehabilitation and self-management strategies. Within group improvements in GAS appear clinically relevant, but without a comparator cannot rule out Hawthorne or natural history effects. | | | | | | | | | |  |  |  |  |  |  |
| [15] | 2020, McAllister, M., et al. | 183 | 3 | 1 | - | - | 1 | - | 0 | 0 | 0 | 14 | Memory Keeper App Usability and Impact Assessment | 1 | Memory Keeper App Usability and Impact Assessment | 1 | 14.1 |
|  |  | Context: Memory Keeper Digital Application. Using the Memory Keeper app to stimulate reminiscences and meaningful engagement with people with dementia in long-term care. The intervention is based on the concept that personalised digital prompts can stimulate reminiscences and improve engagement with people with dementia. Qualitative accounts of increased social engagement, extended visit duration and carer satisfaction. Potentially valuable but anecdotal; magnitude and consistency uncertain. | | | | | | | | | |  |  |  |  |  |  |
| [16] | 2019, Cunningham, S., et al. | 14 | 14 | - | - | 0 | 0 | 0 | 0 | 0 | 0 | 4 | Quality of Life in Alzheimer’s Disease (QoL-AD). | 4 | Self-Assessment Manikin (SAM) | 3 | 9.2 |
|  |  | Context: Memory Tracks Mobile App. This goal focuses on using reminiscence music through a mobile app to improve the wellbeing and quality of life of persons living with dementia, as well as to support their caregivers. Research focused on the potential benefits of music as a trigger to support daily activities for people living with dementia. The study explored the use of music from early childhood as triggers associated with specific daily tasks. Statistically significant but small within subject improvements in valence ( 0.38 on 9 point scale) and physical health (+0.40 on 4 point scale). Clinical importance unclear. | | | | | | | | | |  |  |  |  |  |  |
| [17] | 2019, Lancioni, G.E., et al. | 2 | 21 | - | - | - | - | - | 0 | 0 | 0 | 5 | Smartphone Prompts, Indices of Personal Satisfaction. | 1 | Card or Bottle Responses, Smartphone Prompts, Heart Rate Monitoring. | 1 | 4.15 |
|  |  | Context: Smartphone-Based Stimulation. This goal aims to increase physical activity and personal satisfaction in individuals with advanced Alzheimer's disease through engaging arm movements and cognitive stimulation. Use of smartphone technology to stimulate physical and cognitive activity in Alzheimer's patients. Large within subject gains in simple activity and observable enjoyment; physiologically small to moderate heart rate rise whose clinical importance is unclear. | | | | | | | | | |  |  |  |  |  |  |
| [18] | 2019, Braley, R., et al. | 1 | 15 | - | - | - | - | - | 0 | 0 | 0 | 10 | Smart Home Voice IADL Prompting Response Analysis. | 1 | Smart Home Voice IADL Prompting Response Analysis. | 1 | 5.3 |
|  |  | Context: Smart Home Auto-Prompting System. This goal aims to increase the functional independence of persons with dementia (PWDs) using smart home auto-prompting technology, thereby reducing caregiver burden. The focus is on assisting PWDs in completing instrumental activities of daily living (IADLs) at home. Research focused on understanding how PWDs responded to auto-prompting while performing IADL tasks. The study utilised qualitative methods to analyse behaviours of PWDs interacting with the technology. Auto prompts facilitated completion of some IADL steps, particularly in very mild dementia, and reduced need for human assistance in a subset of tasks. | | | | | | | | | |  |  |  |  |  |  |
| [19] | 2018, Siddiq, K., et al. | 1 | 25 | - | - | - | - | - | 0 | 0 | 0 | 73 | CareD App Usability and Effectiveness evaluation. | 1 | CareD App Usability and Effectiveness evaluation. | 1 | 17.9 |
|  |  | Context: Adoption of CareD Mobile Application. Using the CareD mobile application to provide cognitive support, reminders, and patient tracking to improve the daily functioning and quality of life of dementia patients. The intervention is based on the need for accessible, non-drug treatments for dementia, focusing on cognitive therapies and daily assistance. Usability signals suggest most mild stage users completed simple tasks within 2 min; moderate stage users required longer but still managed (3-5.5 min). Caregivers reported ~80 % satisfaction with app functionality and terminology. | | | | | | | | | |  |  |  |  |  |  |
| [20] | 2016, Hartin, P.J., et al. | 183 | 96 | - | - | - | - | - | 0 | 0 | 0 | 41 | None | 0 | Gray Matters App Usage and Behavioural Change Assessment. | 1 | 23.15 |
|  |  | Context: Gray Matters Mobile App Usage. Goal aimed at reducing the risk of Alzheimer's Disease through behaviour change, focusing on improving vascular health and addressing various risk factors. Research focused on the effectiveness of the Gray Matters app in encouraging and facilitating behaviour change across various domains related to AD risk. The study involved a randomized controlled trial with participants using the app to receive educational material, self-report behaviours, and get feedback on performance. Small within group improvements in surrogate vascular risk factors were associated with higher app engagement, but causal benefit over control remains unquantified. | | | | | | | | | |  |  |  |  |  |  |
| [21] | 2015, Tomori, K., et al. | 91 | 116 | 21 | - | - | - | - | 0 | 0 | 0 | 6 | Aid for Decision-making in Occupation Choice (ADOC) | 1 | Aid for Decision-making in Occupation Choice (ADOC), Mini-Mental State Examination (MMSE) | 2 | 14.55 |
|  |  | Context: Use of ADOC for Activity Selection. Using the Aid for Decision-making in Occupation Choice (ADOC) iPad application to identify and engage in meaningful activities for dementia patients. Investigation into the effectiveness of ADOC in helping dementia patients choose meaningful activities, with a focus on determining a cut-off MMSE score for its use. High diagnostic accuracy (AUC 0.89) suggests ADOC can identify patients (MMSE ≥ 8) who can articulate meaningful activities. | | | | | | | | | |  |  |  |  |  |  |
| [22] | 2015, Norton, M.C., et al. | 183 | Not specified | - | - | - | - | - | 0 | 0 | 0 | 27 | Online Surveys, Smartphone App Usage, Physical Measurements: | 2 | Cognitive Tests, Online Surveys, Smartphone App Usage | 2 | 18.55 |
|  |  | Context: Use of Custom Smartphone Application and Activity Monitor. Implementing a multidomain lifestyle intervention to reduce the risk of Alzheimer's disease in middle-aged individuals through behavioural changes. The intervention is based on evidence linking lifestyle factors with Alzheimer's disease risk, including physical activity, diet, cognitive stimulation, social engagement, stress management, and sleep quality. Preliminary process indicators (engagement, compliance) are encouraging but because no efficacy effect sizes or confidence intervals are reported, net clinical benefit cannot be quantified. | | | | | | | | | |  |  |  |  |  |  |
| [23] | 2009, Adlam, T., et al. | 365 | Not specified | - | - | - | - | - | 1 | 0 | 0 | 17 | Individual Prioritised Problem Assessment (IPPA), Behaviour Monitoring in Smart Homes, Functional Assessment Scale (FAS), Sensor Derived Measures. | 3 | Behaviour Monitoring in Smart Homes, Functional Assessment Scale (FAS), Mini Mental State Examination (MMSE). Sensor Derived Measures | 2 | 31.65 |
|  |  | Context: COACH System. HELPER System. This goal focuses on using smart home technology to enhance the safety and independence of people with dementia. The technology aims to monitor and respond to potential crisis events like falls or sudden illness and assist in daily living activities. Technology was installed and evaluated in smart flats in London and Bristol, demonstrating positive impacts on residents' lives, particularly in improving sleep and overall well-being. Narrative evidence of better sleep and functional gains; unquantified and possibly clinically relevant but based on uncontrolled observation. | | | | | | | | | |  |  |  |  |  |  |
| [24] | 2009, Bewernitz, M.W., et al. | 1 | 11 | - | - | - | - | - | 0 | 0 | 0 | 14 | Machine-Based Prompting Efficacy | 1 | Machine-Based Prompting Efficacy | 1 | 5.4 |
|  |  | Context: Machine-Based Prompting System. This goal focuses on using machine-based prompting to assist individuals with dementia in performing daily living tasks, thereby enhancing their independence, and reducing caregiver burden. The study investigates the use of machine-based prompting to assist in self-care tasks for individuals with dementia, focusing on tasks of varied complexity. It draws on past research indicating the effectiveness of prompting in improving task performance in dementia patients. Across all three tasks participants completed 86 % of steps when prompted (no CIs reported). Baseline completion rates were not provided numerically; authors state performance was “substantially lower” without prompts. | | | | | | | | | |  |  |  |  |  |  |

References

1. Rossetto, F., et al., *A digital health home intervention for people within the Alzheimer's disease continuum: results from the Ability-TelerehABILITation pilot randomized controlled trial.* Ann Med, 2023. **55**(1): p. 1080-1091 DOI: 10.1080/07853890.2023.2185672.

2. Menengiç, K.N., et al., *Effectiveness of motor-cognitive dual-task exercise via telerehabilitation in Alzheimer's disease: An online pilot randomized controlled study.* Clin Neurol Neurosurg, 2022. **223**: p. 107501 DOI: 10.1016/j.clineuro.2022.107501.

3. Kerkhof, Y., et al., *Randomized controlled feasibility study of FindMyApps: first evaluation of a tablet-based intervention to promote self-management and meaningful activities in people with mild dementia.* Disabil Rehabil Assist Technol, 2022. **17**(1): p. 85-99 DOI: 10.1080/17483107.2020.1765420.

4. Dinesen, B., et al., *Use of a Social Robot (LOVOT) for Persons With Dementia: Exploratory Study.* JMIR Rehabil Assist Technol, 2022. **9**(3): p. e36505 DOI: 10.2196/36505.

5. Han, S.S., K. White, and E. Cisek, *A Feasibility Study of Individuals Living at Home with Alzheimer's Disease and Related Dementias: Utilization of Visual Mapping Assistive Technology to Enhance Quality of Life and Reduce Caregiver Burden.* Clin Interv Aging, 2022. **17**: p. 1885-1892 DOI: 10.2147/cia.S387255.

6. Larnyo, E., et al., *Impact of Actual Use Behavior of Healthcare Wearable Devices on Quality of Life: A Cross-Sectional Survey of People with Dementia and Their Caregivers in Ghana.* Healthcare (Basel), 2022. **10**(2) DOI: 10.3390/healthcare10020275.

7. Freytag, J., et al., *Using Wearable Sensors to Measure Goal Achievement in Older Veterans with Dementia.* Sensors (Basel), 2022. **22**(24) DOI: 10.3390/s22249923.

8. Kelleher, J., et al., *Personalized Visual Mapping Assistive Technology to Improve Functional Ability in Persons With Dementia: Feasibility Cohort Study.* JMIR Aging, 2021. **4**(4): p. e28165 DOI: 10.2196/28165.

9. Howard, R., et al., *The effectiveness and cost-effectiveness of assistive technology and telecare for independent living in dementia: a randomised controlled trial.* Age Ageing, 2021. **50**(3): p. 882-890 DOI: 10.1093/ageing/afaa284.

10. Harris, N., et al., *A preliminary evaluation of a client-centred prompting tool for supporting everyday activities in individuals with mild to moderate levels of cognitive impairment due to dementia.* Dementia (London), 2021. **20**(3): p. 867-883 DOI: 10.1177/1471301220911322.

11. Goodall, G., et al., *Supporting identity and relationships amongst people with dementia through the use of technology: a qualitative interview study.* Int J Qual Stud Health Well-being, 2021. **16**(1): p. 1920349 DOI: 10.1080/17482631.2021.1920349.

12. Gall, D., et al., *Self-organizing knowledge management might improve the quality of person-centered dementia care: A qualitative study.* Int J Med Inform, 2020. **139**: p. 104132 DOI: 10.1016/j.ijmedinf.2020.104132.

13. Ferry, F., et al., *Economic costs and health-related quality of life associated with individual specific reminiscence: Results from the InspireD Feasibility Study.* Dementia (London), 2020. **19**(7): p. 2166-2183 DOI: 10.1177/1471301218816814.

14. Øksnebjerg, L., et al., *Self-management and cognitive rehabilitation in early stage dementia - merging methods to promote coping and adoption of assistive technology. A pilot study.* Aging Ment Health, 2020. **24**(11): p. 1894-1903 DOI: 10.1080/13607863.2019.1625302.

15. McAllister, M., et al., *Memory Keeper: A prototype digital application to improve engagement with people with dementia in long-term care (innovative practice).* Dementia (London), 2020. **19**(4): p. 1287-1298 DOI: 10.1177/1471301217737872.

16. Cunningham, S., et al., *Assessing Wellbeing in People Living with Dementia Using Reminiscence Music with a Mobile App (Memory Tracks): A Mixed Methods Cohort Study.* Journal of Healthcare Engineering, 2019. **2019**: p. 8924273 DOI: 10.1155/2019/8924273.

17. Lancioni, G.E., et al., *Smartphone-Based Interventions to Foster Simple Activity and Personal Satisfaction in People With Advanced Alzheimer's Disease.* Am J Alzheimers Dis Other Demen, 2019. **34**(7-8): p. 478-485 DOI: 10.1177/1533317519844144.

18. Braley, R., et al., *Prompting Technology and Persons With Dementia: The Significance of Context and Communication.* Gerontologist, 2019. **59**(1): p. 101-111 DOI: 10.1093/geront/gny071.

19. Siddiq, K., et al., *CareD: Non-Pharmacological Assistance for Dementia Patients.* EAI Endorsed Transactions on Pervasive Health and Technology, 2018. **4**: p. 160073 DOI: 10.4108/eai.13-7-2018.160073.

20. Hartin, P.J., et al., *The Empowering Role of Mobile Apps in Behavior Change Interventions: The Gray Matters Randomized Controlled Trial.* JMIR Mhealth Uhealth, 2016. **4**(3): p. e93 DOI: 10.2196/mhealth.4878.

21. Tomori, K., et al., *Examination of a cut-off score to express the meaningful activity of people with dementia using iPad application (ADOC).* Disabil Rehabil Assist Technol, 2015. **10**(2): p. 126-31 DOI: 10.3109/17483107.2013.871074.

22. Norton, M.C., et al., *The design and progress of a multidomain lifestyle intervention to improve brain health in middle-aged persons to reduce later Alzheimer's disease risk: The Gray Matters randomized trial.* Alzheimers Dement (N Y), 2015. **1**(1): p. 53-62 DOI: 10.1016/j.trci.2015.05.001.

23. Adlam, T., et al., *Implementing Monitoring and Technological Interventions in Smart Homes for People with Dementia - Case Studies*. 2009. 159-182.

24. Bewernitz, M.W., et al., *Feasibility of machine-based prompting to assist persons with dementia.* Assist Technol, 2009. **21**(4): p. 196-207 DOI: 10.1080/10400430903246050.
