# Supplementary material for: Wearable Technology, Smart Home Systems, and Mobile Apps for the Self‑Management of Patient Outcomes in Dementia Care: Systematic Review
Source: J Med Internet Res. 2025 Aug 21;27:e65385. doi: 10.2196/65385 (PMC12411798; doi:10.2196/65385)
Supplement: Multimedia Appendix 5 [file jmir_v27i1e65385_app5.docx]

Appendix 5. National Institute for Health and Care Excellence quality standard 184 subgroup analysis.

[1] [2] [3] [4]

[5] [6] [7] [8]

[9] [10] [11] [12]

[13] [14] [15] [16]

[17] [18] [19] [20]

[21] [22] [23] [24]

References

1. Menengiç, K.N., et al., *Effectiveness of motor-cognitive dual-task exercise via telerehabilitation in Alzheimer's disease: An online pilot randomized controlled study.* Clin Neurol Neurosurg, 2022. **223**: p. 107501 DOI: 10.1016/j.clineuro.2022.107501

2. Gall, D., et al., *Self-organizing knowledge management might improve the quality of person-centered dementia care: A qualitative study.* Int J Med Inform, 2020. **139**: p. 104132 DOI: 10.1016/j.ijmedinf.2020.104132.

3. Norton, M.C., et al., *The design and progress of a multidomain lifestyle intervention to improve brain health in middle-aged persons to reduce later Alzheimer's disease risk: The Gray Matters randomized trial.* Alzheimers Dement (N Y), 2015. **1**(1): p. 53-62 DOI: 10.1016/j.trci.2015.05.001.

4. Rossetto, F., et al., *A digital health home intervention for people within the Alzheimer's disease continuum: results from the Ability-TelerehABILITation pilot randomized controlled trial.* Ann Med, 2023. **55**(1): p. 1080-1091 DOI: 10.1080/07853890.2023.2185672.

5. Øksnebjerg, L., et al., *Self-management and cognitive rehabilitation in early stage dementia - merging methods to promote coping and adoption of assistive technology. A pilot study.* Aging Ment Health, 2020. **24**(11): p. 1894-1903 DOI: 10.1080/13607863.2019.1625302.

6. Goodall, G., et al., *Supporting identity and relationships amongst people with dementia through the use of technology: a qualitative interview study.* Int J Qual Stud Health Well-being, 2021. **16**(1): p. 1920349 DOI: 10.1080/17482631.2021.1920349.

7. Kerkhof, Y., et al., *Randomized controlled feasibility study of FindMyApps: first evaluation of a tablet-based intervention to promote self-management and meaningful activities in people with mild dementia.* Disabil Rehabil Assist Technol, 2022. **17**(1): p. 85-99 DOI: 10.1080/17483107.2020.1765420.

8. Bewernitz, M.W., et al., *Feasibility of machine-based prompting to assist persons with dementia.* Assist Technol, 2009. **21**(4): p. 196-207 DOI: 10.1080/10400430903246050.

9. Howard, R., et al., *The effectiveness and cost-effectiveness of assistive technology and telecare for independent living in dementia: a randomised controlled trial.* Age Ageing, 2021. **50**(3): p. 882-890 DOI: 10.1093/ageing/afaa284.

10. Braley, R., et al., *Prompting Technology and Persons With Dementia: The Significance of Context and Communication.* Gerontologist, 2019. **59**(1): p. 101-111 DOI: 10.1093/geront/gny071.

11. Cunningham, S., et al., *Assessing Wellbeing in People Living with Dementia Using Reminiscence Music with a Mobile App (Memory Tracks): A Mixed Methods Cohort Study.* Journal of Healthcare Engineering, 2019. **2019**: p. 8924273 DOI: 10.1155/2019/8924273.

12. McAllister, M., et al., *Memory Keeper: A prototype digital application to improve engagement with people with dementia in long-term care (innovative practice).* Dementia (London), 2020. **19**(4): p. 1287-1298 DOI: 10.1177/1471301217737872.

13. Ferry, F., et al., *Economic costs and health-related quality of life associated with individual specific reminiscence: Results from the InspireD Feasibility Study.* Dementia (London), 2020. **19**(7): p. 2166-2183 DOI: 10.1177/1471301218816814.

14. Harris, N., et al., *A preliminary evaluation of a client-centred prompting tool for supporting everyday activities in individuals with mild to moderate levels of cognitive impairment due to dementia.* Dementia (London), 2021. **20**(3): p. 867-883 DOI: 10.1177/1471301220911322.

15. Lancioni, G.E., et al., *Smartphone-Based Interventions to Foster Simple Activity and Personal Satisfaction in People With Advanced Alzheimer's Disease.* Am J Alzheimers Dis Other Demen, 2019. **34**(7-8): p. 478-485 DOI: 10.1177/1533317519844144.

16. Han, S.S., K. White, and E. Cisek, *A Feasibility Study of Individuals Living at Home with Alzheimer's Disease and Related Dementias: Utilization of Visual Mapping Assistive Technology to Enhance Quality of Life and Reduce Caregiver Burden.* Clin Interv Aging, 2022. **17**: p. 1885-1892 DOI: 10.2147/cia.S387255.

17. Kelleher, J., et al., *Personalized Visual Mapping Assistive Technology to Improve Functional Ability in Persons With Dementia: Feasibility Cohort Study.* JMIR Aging, 2021. **4**(4): p. e28165 DOI: 10.2196/28165.

18. Dinesen, B., et al., *Use of a Social Robot (LOVOT) for Persons With Dementia: Exploratory Study.* JMIR Rehabil Assist Technol, 2022. **9**(3): p. e36505 DOI: 10.2196/36505.

19. Hartin, P.J., et al., *The Empowering Role of Mobile Apps in Behavior Change Interventions: The Gray Matters Randomized Controlled Trial.* JMIR Mhealth Uhealth, 2016. **4**(3): p. e93 DOI: 10.2196/mhealth.4878.

20. Tomori, K., et al., *Examination of a cut-off score to express the meaningful activity of people with dementia using iPad application (ADOC).* Disabil Rehabil Assist Technol, 2015. **10**(2): p. 126-31 DOI: 10.3109/17483107.2013.871074.

21. Adlam, T., et al., *Implementing Monitoring and Technological Interventions in Smart Homes for People with Dementia - Case Studies*. 2009. 159-182.

22. Larnyo, E., et al., *Impact of Actual Use Behavior of Healthcare Wearable Devices on Quality of Life: A Cross-Sectional Survey of People with Dementia and Their Caregivers in Ghana.* Healthcare (Basel), 2022. **10**(2) DOI: 10.3390/healthcare10020275

23. Freytag, J., et al., *Using Wearable Sensors to Measure Goal Achievement in Older Veterans with Dementia.* Sensors (Basel), 2022. **22**(24) DOI: 10.3390/s22249923

24. Siddiq, K., et al., *CareD: Non-Pharmacological Assistance for Dementia Patients.* EAI Endorsed Transactions on Pervasive Health and Technology, 2018. **4**: p. 160073 DOI: 10.4108/eai.13-7-2018.160073.
