# Supplementary material for: Wearable Technology, Smart Home Systems, and Mobile Apps for the Self‑Management of Patient Outcomes in Dementia Care: Systematic Review
Source: J Med Internet Res. 2025 Aug 21;27:e65385. doi: 10.2196/65385 (PMC12411798; doi:10.2196/65385)
Supplement: Multimedia Appendix 6 [file jmir_v27i1e65385_app6.docx]

#### Appendix 6. Nursing Outcomes Classification subgroup analysis.

The following items are the subgroup identified from NOC as the most relevant to the research questions in this study:

- Functional health
  - Energy maintenance
    - 0007 | Fatigue level [21][6]
    - 0004 | Sleep [7][9][16][17][18][19][21][22][5][6][24]
  - Mobility
    - 0200 | Ambulation [10][7][13][3][17][4][21][5][6]
    - 0202 | Balance [10][17][21]
    - 0212 | Coordinated movement [10][17][4][21]
    - 0222 | Gait [10][17][21]
    - 0208 | Mobility [13][3][14][17][21][6]
    - 0210 | Transfer performance [17][21]
  - Self-care
    - 0313 | Self-care behavior [10][11][12][2][13][3][14][16][17][20][21][22][23][6][24]
    - 0300 | 370885003 Self-care behavior: activities of daily living [10][11][12][2][7][13][3][14][16][17][20][21][6][24]
    - 0301 | Self-care behavior: bathing [13][17][21]
    - 0302 | Self-care behavior: dressing [13][17][21][24]
    - 0303 | Self-care behavior: eating [14][17][21][22][23][5][6][24]
    - 0305 | Self-care behavior: hygiene [17][21][24]
    - 0306 | Self-care behavior: instrumental activities of daily living [7][14][17][20][21][23][6]
    - 0307 | Self-care behavior: nonparenteral medication [13][17][21]
    - 0308 | Self-care behavior: oral hygiene [17][21][24]
    - 0310 | Self-care behavior: toileting [13][17][21]
- Physiological health
  - Digestion and nutrition
    - 1014 | Appetite [17][5]
    - 1015 | Gastrointestinal function [17]
    - 1004 | Nutritional status [17][22][5]
    - 1007 | Nutritional status: energy [17][22][5]
    - 1008 | Nutritional status: food and fluid intake [17][22][5]
    - 1009 | Nutritional status: nutritional intake [17][22][5]
    - 1010 | 405035003 Swallowing status [17]
  - Elimination
    - 0500 | Bowel continence [17]
    - 0502 | Urinary continence [17]
  - Fluid and electrolytes
    - 0606 | Electrolyte balance [17]
    - 0601 | Fluid balance [17]
    - 0602 | Hydration [17]
  - Metabolic regulation
    - 0802 | Vital signs [17]
    - 1006 | Weight: body mass [17][22][5]
  - Neurocognitive
    - 0900 | Cognition [1][10][11][8][12][2][7][13][3][14][9][15][16][17][18][19][4][20][21][22][23][5][6][24]
    - 0901 | Cognition orientation [17][21]
    - 0902 | Communication [1][11][8][7][14][9][15][16][17][18][19][4][20][21][22][23][6][24]
    - 0905 | Concentration [1][10][17][21][22]
    - 0906 | Decision-making [17][21][22]
    - 0916 | Delirium level [17]
    - 0920 | Dementia level [1][10][11][8][12][2][7][13][3][14][9][15][16][17][18][19][4][20][21][23][6][24]
    - 0915 | Hyperactivity level [17]
    - 0907 | Information processing [1][10][11][17][20][21][22][6][24]
    - 0908 | Memory [1][10][11][8][12][2][7][13][3][14][9][21]
- Psychosocial health
  - Psychological well-being
    - 1214 | Agitation level
    - 1211 | Anxiety level [10][16][17]
    - 1208 | Depression level [10][13][17]
    - 1210 | Fear level [17]
    - 1203 | Loneliness severity [17][18]
    - 1204 | Mood equilibrium [13][17]
    - 1209 | Motivation [11][17][21][22][5][6][24]
    - 1217 | Panic level
    - 1202 | Personal identity [9][17][21]
    - 1215 | Self-awareness [11][9][17][21][22]
    - 1216 | Social anxiety level
    - 1212 | Stress level [16][17][21][22][5][6][24]
    - 1206 | Will to live [17]
  - Psychosocial adaptation
    - 1300 | Acceptance: health status [17]
    - 1302 | Coping [11][16][17][18][21][22][5][6][24]
    - 1304 | Grief resolution
    - 1310 | Guilt resolution
    - 1309 | Personal resilience [11][17][21]
    - 1305 | Psychosocial adjustment life change [17][21]
    - 1311 | Relocation adaptation
  - Self-control
    - 1400 | Abusive behavior
    - 1401 | Aggression self-restraint
    - 1410 | Anger self-restraint
    - 1402 | Anxiety self-control [17]
    - 1409 | Depression self-control [17]
    - 1403 | Distorted thought self-control
    - 1404 | Fear self-control [17]
    - 1405 | Impulse self-control
    - 1412 | Panic self-control
    - 1414 | Self-harm restraint
  - Social interaction
    - 1503 | Social involvement [11][8][2][13][9][15][16][17][18][21][22][5][6]
    - 1504 | Social support [11][8][2][9][15][16][17][18][21][22][5][6]
- Health knowledge and behavior condition
  - Health behavior
    - 1600 | Adherence behavior [1][10][11][2][17][21][22][5]
    - 1640 | Adherence behavior: clinical condition [1][10][11][2][17][21][22][5]
    - 1621 | Adherence behavior: healthy diet [17][21][22][5]
    - 1632 | Adherence behavior: prescribed activity [1][10][11][17][21][22][5]
    - 1622 | Adherence behavior: prescribed diet [17][21][22][5]
    - 1623 | Adherence behavior: prescribed medication [17][21]
    - 1633 | Exercise participation [1][10][11][7][17][21][22][5]
    - 1604 | Leisure participation [11][16][17][18][21][22][5]
    - 1605 | Pain control [17]
    - 1606 | Participation in health care decisions [7][15][16][17][21][22]
    - 1638 | Patient engagement behavior [1][11][7][15][16][17][21][22]
    - 1614 | Personal autonomy [11][15][16][17][21][22]
    - 1613 | Self-direction of care [1][11][15][16][17][21][22]
    - 1639 | Self-direction of instrumental activities of daily living [17][20][21][22]
    - 1642 | Sleep enhancement behavior [17][21][22][5]
  - Knowledge health condition
    - 1851 | Knowledge: dementia management [1][10][11][13][15][16][17][20][21]
  - Knowledge health promotion
    - 1867 | Knowledge: diagnostic and therapeutic procedures
    - 1804 | Knowledge: energy conservation [17][22]
    - 1828 | Knowledge: fall prevention [17]
    - 1805 | Knowledge: health behavior [1][11][15][16][17][21][22][5]
    - 1806 | Knowledge: health resources [11][17][21][22]
    - 1854 | Knowledge: healthy diet [17][21][22][5]
    - 1855 | Knowledge: healthy lifestyle [11][17][21][22][5]
    - 1808 | Knowledge: medication [17][21]
    - 1843 | Knowledge: pain management [17]
    - 1809 | Knowledge: pain personal safety [17]
    - 1862 | Knowledge: stress management [17][21][22][5]
  - Risk control
    - 1902 | Risk control [17]
    - 1903 | Risk control: alcohol use [17]
    - 1935 | Risk control: aspiration [17]
    - 1937 | Risk control: dehydration [17]
    - 1938 | Risk control: environmental hazards [17]
    - 1939 | Risk control: falls [10][17]
  - Safety
    - 1919 | Elopement occurrence
    - 1920 | Elopement propensity risk
    - 1909 | Fall prevention behavior [17]
    - 1912 | Falls occurrence
    - 1911 | Personal safety behavior [17][21]
    - 1934 | Safe health care environment [17]
    - 1910 | Safe home environment [17]
    - 1926 | Safe wandering [21]
- Health and life quality
  - Health status
    - 2006 | Personal health status [10][11][2][16][17][21][22]
  - Symptom status
    - 2109 | Discomfort level [17]
    - 2101 | Pain: disruptive effects [17]
    - 2102 | Pain level [17]
    - 2119 | Sleep disruptive severity [16][17][21]
    - 2003 | Distress severity [17]
    - 2103 | Symptom severity [17][21]
  - Perceived health and life situation
    - 2008 | Comfort status [16][17][21]
    - 2009 | Comfort status: environment [17][21]
    - 2010 | Comfort status: physical [16][17][21]
    - 2011 | Comfort status: psychospiritual [16][17][21]
    - 2012 | Comfort status: sociocultural [17][21]
    - 2002 | Personal well-being [11][16][17][21][22]
    - 2000 | Quality of life [10][11][2][15][16][17][21][22]
    - 2001 | Spiritual health [17]
- Family health
  - Family well-being
    - 2600 | Family coping [11][16][17][18][21]
    - 2602 | Family functioning [11][16][17][18][21]
    - 2603 | Family integrity [17][18][21]
    - 2212 | Family performance in dementia care [1][15][16][17][18][20][21]
    - 2608 | Family resilience [11][17][18][21]

References

1. Rossetto, F., et al., *A digital health home intervention for people within the Alzheimer's disease continuum: results from the Ability-TelerehABILITation pilot randomized controlled trial.* Ann Med, 2023. **55**(1): p. 1080-1091 DOI: 10.1080/07853890.2023.2185672.

2. Larnyo, E., et al., *Impact of Actual Use Behavior of Healthcare Wearable Devices on Quality of Life: A Cross-Sectional Survey of People with Dementia and Their Caregivers in Ghana.* Healthcare (Basel), 2022. **10**(2) DOI: 10.3390/healthcare10020275.

3. Howard, R., et al., *The effectiveness and cost-effectiveness of assistive technology and telecare for independent living in dementia: a randomised controlled trial.* Age Ageing, 2021. **50**(3): p. 882-890 DOI: 10.1093/ageing/afaa284.

4. Lancioni, G.E., et al., *Smartphone-Based Interventions to Foster Simple Activity and Personal Satisfaction in People With Advanced Alzheimer's Disease.* Am J Alzheimers Dis Other Demen, 2019. **34**(7-8): p. 478-485 DOI: 10.1177/1533317519844144.

5. Norton, M.C., et al., *The design and progress of a multidomain lifestyle intervention to improve brain health in middle-aged persons to reduce later Alzheimer's disease risk: The Gray Matters randomized trial.* Alzheimers Dement (N Y), 2015. **1**(1): p. 53-62 DOI: 10.1016/j.trci.2015.05.001.

6. Adlam, T., et al., *Implementing Monitoring and Technological Interventions in Smart Homes for People with Dementia - Case Studies*. 2009. 159-182.

7. Freytag, J., et al., *Using Wearable Sensors to Measure Goal Achievement in Older Veterans with Dementia.* Sensors (Basel), 2022. **22**(24) DOI: 10.3390/s22249923.

8. Dinesen, B., et al., *Use of a Social Robot (LOVOT) for Persons With Dementia: Exploratory Study.* JMIR Rehabil Assist Technol, 2022. **9**(3): p. e36505 DOI: 10.2196/36505.

9. Goodall, G., et al., *Supporting identity and relationships amongst people with dementia through the use of technology: a qualitative interview study.* Int J Qual Stud Health Well-being, 2021. **16**(1): p. 1920349 DOI: 10.1080/17482631.2021.1920349.

10. Menengiç, K.N., et al., *Effectiveness of motor-cognitive dual-task exercise via telerehabilitation in Alzheimer's disease: An online pilot randomized controlled study.* Clin Neurol Neurosurg, 2022. **223**: p. 107501 DOI: 10.1016/j.clineuro.2022.107501.

11. Kerkhof, Y., et al., *Randomized controlled feasibility study of FindMyApps: first evaluation of a tablet-based intervention to promote self-management and meaningful activities in people with mild dementia.* Disabil Rehabil Assist Technol, 2022. **17**(1): p. 85-99 DOI: 10.1080/17483107.2020.1765420.

12. Han, S.S., K. White, and E. Cisek, *A Feasibility Study of Individuals Living at Home with Alzheimer's Disease and Related Dementias: Utilization of Visual Mapping Assistive Technology to Enhance Quality of Life and Reduce Caregiver Burden.* Clin Interv Aging, 2022. **17**: p. 1885-1892 DOI: 10.2147/cia.S387255.

13. Kelleher, J., et al., *Personalized Visual Mapping Assistive Technology to Improve Functional Ability in Persons With Dementia: Feasibility Cohort Study.* JMIR Aging, 2021. **4**(4): p. e28165 DOI: 10.2196/28165.

14. Harris, N., et al., *A preliminary evaluation of a client-centred prompting tool for supporting everyday activities in individuals with mild to moderate levels of cognitive impairment due to dementia.* Dementia (London), 2021. **20**(3): p. 867-883 DOI: 10.1177/1471301220911322.

15. Gall, D., et al., *Self-organizing knowledge management might improve the quality of person-centered dementia care: A qualitative study.* Int J Med Inform, 2020. **139**: p. 104132 DOI: 10.1016/j.ijmedinf.2020.104132.

16. Ferry, F., et al., *Economic costs and health-related quality of life associated with individual specific reminiscence: Results from the InspireD Feasibility Study.* Dementia (London), 2020. **19**(7): p. 2166-2183 DOI: 10.1177/1471301218816814.

17. Øksnebjerg, L., et al., *Self-management and cognitive rehabilitation in early stage dementia - merging methods to promote coping and adoption of assistive technology. A pilot study.* Aging Ment Health, 2020. **24**(11): p. 1894-1903 DOI: 10.1080/13607863.2019.1625302.

18. McAllister, M., et al., *Memory Keeper: A prototype digital application to improve engagement with people with dementia in long-term care (innovative practice).* Dementia (London), 2020. **19**(4): p. 1287-1298 DOI: 10.1177/1471301217737872.

19. Cunningham, S., et al., *Assessing Wellbeing in People Living with Dementia Using Reminiscence Music with a Mobile App (Memory Tracks): A Mixed Methods Cohort Study.* Journal of Healthcare Engineering, 2019. **2019**: p. 8924273 DOI: 10.1155/2019/8924273.

20. Braley, R., et al., *Prompting Technology and Persons With Dementia: The Significance of Context and Communication.* Gerontologist, 2019. **59**(1): p. 101-111 DOI: 10.1093/geront/gny071.

21. Siddiq, K., et al., *CareD: Non-Pharmacological Assistance for Dementia Patients.* EAI Endorsed Transactions on Pervasive Health and Technology, 2018. **4**: p. 160073 DOI: 10.4108/eai.13-7-2018.160073.

22. Hartin, P.J., et al., *The Empowering Role of Mobile Apps in Behavior Change Interventions: The Gray Matters Randomized Controlled Trial.* JMIR Mhealth Uhealth, 2016. **4**(3): p. e93 DOI: 10.2196/mhealth.4878.

23. Tomori, K., et al., *Examination of a cut-off score to express the meaningful activity of people with dementia using iPad application (ADOC).* Disabil Rehabil Assist Technol, 2015. **10**(2): p. 126-31 DOI: 10.3109/17483107.2013.871074.

24. Bewernitz, M.W., et al., *Feasibility of machine-based prompting to assist persons with dementia.* Assist Technol, 2009. **21**(4): p. 196-207 DOI: 10.1080/10400430903246050.
