# Supplementary material for: Wearable Technology, Smart Home Systems, and Mobile Apps for the Self‑Management of Patient Outcomes in Dementia Care: Systematic Review
Source: J Med Internet Res. 2025 Aug 21;27:e65385. doi: 10.2196/65385 (PMC12411798; doi:10.2196/65385)
Supplement: Multimedia Appendix 8 [file jmir_v27i1e65385_app8.docx]

Appendix 8. Critical Appraisal Skills Program traffic light checklist.

Randomised controlled trial

1 = Did the study address a clearly focused research question?  2 = Was the assignment of participants to interventions randomised?  3 = Were all participants who entered the study accounted for at its conclusion?  4a = Were the participants ‘blind’ to intervention they were given?  4b = Were the investigators ‘blind’ to the intervention they were giving to participants?  4c = • Were the people assessing/analysing outcome/s ‘blinded’?  5 = Were the study groups similar at the start of the randomised controlled trial?  6 = Apart from the experimental intervention, did each study group receive the same level of care (that is, were they treated equally)?  7 = Were the effects of intervention reported comprehensively?  8 = Was the precision of the estimate of the intervention or treatment effect reported?  9 = Do the benefits of the experimental intervention outweigh the harms and costs?  10 = Can the results be applied to your local population/in your context?  11 = Would the experimental intervention provide greater value to the people in your care than any of the existing interventions?

| **Authors** | **1** | **2** | **3** | **4a** | **4b** | **4c** | **5** | **6** | **7** | **8** | **9** | **10** | **11** |
| --- | --- | --- | --- | --- | --- | --- | --- | --- | --- | --- | --- | --- | --- |
| Menengi Ç, K.N., et al. [1] | Yes | Yes | Yes | Can’t tell | Can’t tell | Can’t tell | Yes | Yes | Yes | Can’t tell | Yes | Can’t tell | Yes |
| Norton, M.C., et al. [2] | Yes | Yes | Can’t tell | No | No | Yes | Yes | No | Can’t tell | Can’t tell | Can’t tell | Can’t tell | Can’t tell |
| Rossetto, F., et al. [3] | Yes | Yes | Yes | No | Yes | Yes | Yes | No | Yes | Yes | Can’t tell | Can’t tell | Yes |
| Howard, R., et al. [4] | Yes | Yes | Yes | No | Can’t tell | Can’t tell | Yes | Can’t tell | Yes | Yes | No | Can’t tell | No |
| Hartin, P.J., et al. [5] | Yes | Yes | Yes | No | Can’t tell | Can’t tell | Yes | Can’t tell | Yes | Can’t tell | Yes | Can’t tell | Can’t tell |
| Kerkhof, Y., et al. [8] | Yes | Yes | Yes | No | No | Yes | Can’t tell | Yes | Yes | Can't tell | Can't tell | Yes | Can't tell |

Qualitative Research

1 = Was there a clear statement of the aims of the research?  2 = Is a qualitative methodology appropriate?  3 = Was the research design appropriate to address the aims of the research?  4 = Was the recruitment strategy appropriate to the aims of the research?  5 = Was the data collected in a way that addressed the research issue?  6 = Has the relationship between researcher and participants been adequately considered?  7 = Have ethical issues been taken into consideration?  8 = Was the data analysis sufficiently rigorous?  9 = Is there a clear statement of findings?  10 = How valuable is the research?

| **Authors** | **1** | **2** | **3** | **4** | **5** | **6** | **7** | **8** | **9** | **10** |
| --- | --- | --- | --- | --- | --- | --- | --- | --- | --- | --- |
| Gall, D., et al. [6] | Yes | Yes | Yes | Yes | Yes | Can’t tell | Yes | Yes | Yes | Yes |
| Goodall, G., et al. [7] | Yes | Yes | Yes | Yes | Yes | Yes | Yes | Yes | Yes | Yes |
| Braley, R., et al. [9] | Yes | Yes | Yes | Yes | Yes | Yes | Yes | Yes | Yes | Yes |
| Cunningham, S., et al. [10] | Yes | Yes | Yes | Yes | Yes | Can’t tell | Yes | Yes | Yes | Yes |
| McAllister, M., et al. [11] | Yes | Yes | Yes | Yes | Yes | Can’t tell | Yes | Yes | Yes | Yes |
| Dinesen, B., et al. [12] | Yes | Yes | Yes | Yes | Yes | Yes | Yes | Yes | Yes | Yes |

Cohort Studies

1 = Did the study address a clearly focused issue?  2 = Was the cohort recruited in an acceptable way?  3 = Was the exposure accurately measured to minimise bias?  4 = Was the outcome accurately measured to minimise bias?  5a = Have the authors identified all important confounding factors?  5b = Have they taken account of the confounding factors in the design and/or analysis?  6a = Was the follow up of subjects complete enough?  6b = Was the follow up of subjects long enough?  7 = What are the results of this study?  8 = How precise are the results?  9 = Do you believe the results?  10 = Can the results be applied to the local population?  11 = Do the results of this study fit with other available evidence?

| **Authors** | **1** | **2** | **3** | **4** | **5a** | **5b** | **6a** | **6b** | **7** | **8** | **9** | **10** | **11** |
| --- | --- | --- | --- | --- | --- | --- | --- | --- | --- | --- | --- | --- | --- |
| Øksnebjerg, L., et al. [13] | Yes | Can’t tell | Yes | Yes | No | Can’t tell | Yes | Can’t tell | Yes | Can’t tell | Yes | Can’t tell | Yes |
| Bewernitz, M.W., et al. [14] | Yes | Yes | Yes | Yes | Can’t tell | Yes | Yes | Can’t tell | Yes | Yes | Yes | Yes | Yes |
| Ferry, F., et al. [15] | Yes | Can’t tell | Yes | Yes | Can’t tell | Can’t tell | Can’t tell | No | Yes | Can’t tell | Can’t tell | Can’t tell | Yes |
| Harris, N., et al. [16] | Yes | Yes | Yes | Yes | Can’t tell | Can’t tell | Yes | Yes | Yes | Can’t tell | Yes | Can’t tell | Can’t tell |
| Lancioni, G.E., et al. [17] | Yes | Yes | Yes | Yes | Can’t tell | Can’t tell | Yes | Yes | Yes | Can’t tell | Yes | Can’t tell | Can’t tell |
| Han, S.S., K. White, and E. Cisek [18] | Yes | Yes | Yes | Yes | Can’t tell | Yes | Yes | Yes | Yes | Can’t tell | Yes | Can’t tell | Yes |
| Kelleher, J., et al. [19] | Yes | Can’t tell | Yes | Yes | Can’t tell | Can’t tell | Yes | Yes | Yes | Can’t tell | Yes | Can’t tell | Can’t tell |
| Tomori, K., et al. [20] | Yes | Can’t tell | Yes | Yes | Can’t tell | Can’t tell | Can’t tell | Can’t tell | Yes | Yes | Yes | Can’t tell | Can’t tell |
| Adlam, T., et al. [21] | Yes | Can’t tell | Yes | Yes | Yes | Yes | Yes | Yes | Yes | Yes | Yes | Yes | Yes |
| Larnyo, E., et al. [22] | Yes | Yes | Yes | Yes | Can’t tell | Can’t tell | Can’t tell | Can’t tell | Yes | Can’t tell | Can’t tell | Can’t tell | Can’t tell |
| Freytag, J., et al. [23] | Yes | Yes | Yes | Yes | Can’t tell | Can’t tell | Yes | Yes | Yes | Can’t tell | Yes | Can’t tell | Can’t tell |
| Siddiq, K., et al. [24] | Yes | Can’t tell | Yes | Yes | Can’t tell | Can’t tell | Can’t tell | Can’t tell | Yes | Can’t tell | Yes | Yes | Can’t tell |

References

1. Menengiç, K.N., et al., *Effectiveness of motor-cognitive dual-task exercise via telerehabilitation in Alzheimer's disease: An online pilot randomized controlled study.* Clin Neurol Neurosurg, 2022. **223**: p. 107501 DOI: 10.1016/j.clineuro.2022.107501.

2. Norton, M.C., et al., *The design and progress of a multidomain lifestyle intervention to improve brain health in middle-aged persons to reduce later Alzheimer's disease risk: The Gray Matters randomized trial.* Alzheimers Dement (N Y), 2015. **1**(1): p. 53-62 DOI: 10.1016/j.trci.2015.05.001.

3. Rossetto, F., et al., *A digital health home intervention for people within the Alzheimer's disease continuum: results from the Ability-TelerehABILITation pilot randomized controlled trial.* Ann Med, 2023. **55**(1): p. 1080-1091 DOI: 10.1080/07853890.2023.2185672.

4. Howard, R., et al., *The effectiveness and cost-effectiveness of assistive technology and telecare for independent living in dementia: a randomised controlled trial.* Age Ageing, 2021. **50**(3): p. 882-890 DOI: 10.1093/ageing/afaa284.

5. Hartin, P.J., et al., *The Empowering Role of Mobile Apps in Behavior Change Interventions: The Gray Matters Randomized Controlled Trial.* JMIR Mhealth Uhealth, 2016. **4**(3): p. e93 DOI: 10.2196/mhealth.4878.

6. Gall, D., et al., *Self-organizing knowledge management might improve the quality of person-centered dementia care: A qualitative study.* Int J Med Inform, 2020. **139**: p. 104132 DOI: 10.1016/j.ijmedinf.2020.104132.

7. Goodall, G., et al., *Supporting identity and relationships amongst people with dementia through the use of technology: a qualitative interview study.* Int J Qual Stud Health Well-being, 2021. **16**(1): p. 1920349 DOI: 10.1080/17482631.2021.1920349.

8. Kerkhof, Y., et al., *Randomized controlled feasibility study of FindMyApps: first evaluation of a tablet-based intervention to promote self-management and meaningful activities in people with mild dementia.* Disabil Rehabil Assist Technol, 2022. **17**(1): p. 85-99 DOI: 10.1080/17483107.2020.1765420.

9. Braley, R., et al., *Prompting Technology and Persons With Dementia: The Significance of Context and Communication.* Gerontologist, 2019. **59**(1): p. 101-111 DOI: 10.1093/geront/gny071.

10. Cunningham, S., et al., *Assessing Wellbeing in People Living with Dementia Using Reminiscence Music with a Mobile App (Memory Tracks): A Mixed Methods Cohort Study.* Journal of Healthcare Engineering, 2019. **2019**: p. 8924273 DOI: 10.1155/2019/8924273.

11. McAllister, M., et al., *Memory Keeper: A prototype digital application to improve engagement with people with dementia in long-term care (innovative practice).* Dementia (London), 2020. **19**(4): p. 1287-1298 DOI: 10.1177/1471301217737872.

12. Dinesen, B., et al., *Use of a Social Robot (LOVOT) for Persons With Dementia: Exploratory Study.* JMIR Rehabil Assist Technol, 2022. **9**(3): p. e36505 DOI: 10.2196/36505.

13. Øksnebjerg, L., et al., *Self-management and cognitive rehabilitation in early stage dementia - merging methods to promote coping and adoption of assistive technology. A pilot study.* Aging Ment Health, 2020. **24**(11): p. 1894-1903 DOI: 10.1080/13607863.2019.1625302.

14. Bewernitz, M.W., et al., *Feasibility of machine-based prompting to assist persons with dementia.* Assist Technol, 2009. **21**(4): p. 196-207 DOI: 10.1080/10400430903246050.

15. Ferry, F., et al., *Economic costs and health-related quality of life associated with individual specific reminiscence: Results from the InspireD Feasibility Study.* Dementia (London), 2020. **19**(7): p. 2166-2183 DOI: 10.1177/1471301218816814.

16. Harris, N., et al., *A preliminary evaluation of a client-centred prompting tool for supporting everyday activities in individuals with mild to moderate levels of cognitive impairment due to dementia.* Dementia (London), 2021. **20**(3): p. 867-883 DOI: 10.1177/1471301220911322.

17. Lancioni, G.E., et al., *Smartphone-Based Interventions to Foster Simple Activity and Personal Satisfaction in People With Advanced Alzheimer's Disease.* Am J Alzheimers Dis Other Demen, 2019. **34**(7-8): p. 478-485 DOI: 10.1177/1533317519844144.

18. Han, S.S., K. White, and E. Cisek, *A Feasibility Study of Individuals Living at Home with Alzheimer's Disease and Related Dementias: Utilization of Visual Mapping Assistive Technology to Enhance Quality of Life and Reduce Caregiver Burden.* Clin Interv Aging, 2022. **17**: p. 1885-1892 DOI: 10.2147/cia.S387255.

19. Kelleher, J., et al., *Personalized Visual Mapping Assistive Technology to Improve Functional Ability in Persons With Dementia: Feasibility Cohort Study.* JMIR Aging, 2021. **4**(4): p. e28165 DOI: 10.2196/28165.

20. Tomori, K., et al., *Examination of a cut-off score to express the meaningful activity of people with dementia using iPad application (ADOC).* Disabil Rehabil Assist Technol, 2015. **10**(2): p. 126-31 DOI: 10.3109/17483107.2013.871074.

21. Adlam, T., et al., *Implementing Monitoring and Technological Interventions in Smart Homes for People with Dementia - Case Studies*. 2009. 159-182.

22. Larnyo, E., et al., *Impact of Actual Use Behavior of Healthcare Wearable Devices on Quality of Life: A Cross-Sectional Survey of People with Dementia and Their Caregivers in Ghana.* Healthcare (Basel), 2022. **10**(2) DOI: 10.3390/healthcare10020275.

23. Freytag, J., et al., *Using Wearable Sensors to Measure Goal Achievement in Older Veterans with Dementia.* Sensors (Basel), 2022. **22**(24) DOI: 10.3390/s22249923.

24. Siddiq, K., et al., *CareD: Non-Pharmacological Assistance for Dementia Patients.* EAI Endorsed Transactions on Pervasive Health and Technology, 2018. **4**: p. 160073 DOI: 10.4108/eai.13-7-2018.160073.
