# Supplementary material for: Wearable Technology, Smart Home Systems, and Mobile Apps for the Self‑Management of Patient Outcomes in Dementia Care: Systematic Review
Source: J Med Internet Res. 2025 Aug 21;27:e65385. doi: 10.2196/65385 (PMC12411798; doi:10.2196/65385)
Supplement: Multimedia Appendix 9 [file jmir_v27i1e65385_app9.docx]

| **Section and Topic** | **Item #** | **Checklist item** | **Location where item is reported** |
| --- | --- | --- | --- |
| **TITLE** | | |  |
| Title | 1 | Identify the report as a systematic review. | P1 > Title |
| **ABSTRACT** | | |  |
| Abstract | 2 | See the PRISMA 2020 for Abstracts checklist. | P2 > Abstract (5 subsubsection) |
| **INTRODUCTION** | | |  |
| Rationale | 3 | Describe the rationale for the review in the context of existing knowledge. | P3 >Introduction |
| Objectives | 4 | Provide an explicit statement of the objective(s) or question(s) the review addresses. | P5 Introduction > Aims and Objectives > Research Questions |
| **METHODS** | | |  |
| Eligibility criteria | 5 | Specify the inclusion and exclusion criteria for the review and how studies were grouped for the syntheses. | P6 Methods > Eligibility Criteria |
| Information sources | 6 | Specify all databases, registers, websites, organisations, reference lists and other sources searched or consulted to identify studies. Specify the date when each source was last searched or consulted. | P7 Methods > Search Strategy AND Abstract > Methods |
| Search strategy | 7 | Present the full search strategies for all databases, registers and websites, including any filters and limits used. | P33 Appendix > Appendices  Table 1. Search Strategy breakdown |
| Selection process | 8 | Specify the methods used to decide whether a study met the inclusion criteria of the review, including how many reviewers screened each record and each report retrieved, whether they worked independently, and if applicable, details of automation tools used in the process. | P7 Methods > Selections Process AND P7 Methods > Extracted Date from Studies |
| Data collection process | 9 | Specify the methods used to collect data from reports, including how many reviewers collected data from each report, whether they worked independently, any processes for obtaining or confirming data from study investigators, and if applicable, details of automation tools used in the process. | P7 Methods > Extracted Date from Studies AND P9 Methods > Data Synthesis |
| Data items | 10a | List and define all outcomes for which data were sought. Specify whether all results that were compatible with each outcome domain in each study were sought (e.g. for all measures, time points, analyses), and if not, the methods used to decide which results to collect. | P8 Methods > Extracted Data from Studies |
|  | 10b | List and define all other variables for which data were sought (e.g. participant and intervention characteristics, funding sources). Describe any assumptions made about any missing or unclear information. | P8 Methods > Extracted Data from Studies |
| Study risk of bias assessment | 11 | Specify the methods used to assess risk of bias in the included studies, including details of the tool(s) used, how many reviewers assessed each study and whether they worked independently, and if applicable, details of automation tools used in the process. | P10 Methods > Data Synthesis |
| Effect measures | 12 | Specify for each outcome the effect measure(s) (e.g. risk ratio, mean difference) used in the synthesis or presentation of results. | P10 Methods > Data Synthesis |
| Synthesis methods | 13a | Describe the processes used to decide which studies were eligible for each synthesis (e.g. tabulating the study intervention characteristics and comparing against the planned groups for each synthesis (item #5)). | P10 Methods > Data Synthesis |
|  | 13b | Describe any methods required to prepare the data for presentation or synthesis, such as handling of missing summary statistics, or data conversions. | P10 Methods > Data Synthesis |
|  | 13c | Describe any methods used to tabulate or visually display results of individual studies and syntheses. | P10 Methods > Data Synthesis |
|  | 13d | Describe any methods used to synthesize results and provide a rationale for the choice(s). If meta-analysis was performed, describe the model(s), method(s) to identify the presence and extent of statistical heterogeneity, and software package(s) used. | P10 Methods > Data Synthesis |
|  | 13e | Describe any methods used to explore possible causes of heterogeneity among study results (e.g. subgroup analysis, meta-regression). | P10 Methods > Data Synthesis |
|  | 13f | Describe any sensitivity analyses conducted to assess robustness of the synthesized results. | P10 Methods > Data Synthesis |
| Reporting bias assessment | 14 | Describe any methods used to assess risk of bias due to missing results in a synthesis (arising from reporting biases). | P10 Methods > Data Synthesis  Appendix MMAT and CASP |
| Certainty assessment | 15 | Describe any methods used to assess certainty (or confidence) in the body of evidence for an outcome. | P10 Methods > Extracted Data from Studies, Appendix Pugh Matrix |
| **RESULTS** | | |  |
| Study selection | 16a | Describe the results of the search and selection process, from the number of records identified in the search to the number of studies included in the review, ideally using a flow diagram. | P11 Findings and Results |
|  | 16b | Cite studies that might appear to meet the inclusion criteria, but which were excluded, and explain why they were excluded. | P46 Appendix > Table 2. Search exclusion decision table. |
| Study characteristics | 17 | Cite each included study and present its characteristics. | P12-22 Results  and P47 Appendix Table 3. Pugh Matrix feature comparison table |
| Risk of bias in studies | 18 | Present assessments of risk of bias for each included study. | P21 Figure 10. Summary of integrated study quality and risk of bias assessment |
| Results of individual studies | 19 | For all outcomes, present, for each study: (a) summary statistics for each group (where appropriate) and (b) an effect estimate and its precision (e.g. confidence/credible interval), ideally using structured tables or plots. | P12-22 Results and P47 Appendix Table 3. Pugh Matrix feature comparison table |
| Results of syntheses | 20a | For each synthesis, briefly summarise the characteristics and risk of bias among contributing studies. | P21 Figure 10. Summary of integrated study quality and risk of bias assessment |
|  | 20b | Present results of all statistical syntheses conducted. If meta-analysis was done, present for each the summary estimate and its precision (e.g. confidence/credible interval) and measures of statistical heterogeneity. If comparing groups, describe the direction of the effect. | P12-22 Results and P47 Appendix Table 3. Pugh Matrix feature comparison table |
|  | 20c | Present results of all investigations of possible causes of heterogeneity among study results. | P19 > Findings and Results > 5. Study Design Quality and Appendix Table 3. Pugh Matrix feature comparison table |
|  | 20d | Present results of all sensitivity analyses conducted to assess the robustness of the synthesized results. | P19 > Findings and Results > 5. Study Design Quality and Appendix: MMAT, CASP and Table 3. Pugh Matrix feature comparison table |
| Reporting biases | 21 | Present assessments of risk of bias due to missing results (arising from reporting biases) for each synthesis assessed. | P19 > Findings and Results > 5. Study Design Quality |
| Certainty of evidence | 22 | Present assessments of certainty (or confidence) in the body of evidence for each outcome assessed. | P47 Appendix Table 3. Pugh Matrix feature comparison table |
| **DISCUSSION** | | |  |
| Discussion | 23a | Provide a general interpretation of the results in the context of other evidence. | P23 > Discussion > Principle Results |
|  | 23b | Discuss any limitations of the evidence included in the review. | P27 > Discussion > Principle Results >5. Study Design Quality |
|  | 23c | Discuss any limitations of the review processes used. | P28 > Discussion > Principle Results > Limitations |
|  | 23d | Discuss implications of the results for practice, policy, and future research. | P28 > Discussion > Principle Results > Conclusions |
| **OTHER INFORMATION** | | |  |
| Registration and protocol | 24a | Provide registration information for the review, including register name and registration number, or state that the review was not registered. | P2 Abstract > Methods AND P5 Methods |
|  | 24b | Indicate where the review protocol can be accessed, or state that a protocol was not prepared. | P2 Abstract > Methods AND P5 Methods |
|  | 24c | Describe and explain any amendments to information provided at registration or in the protocol. | P6 Methods |
| Support | 25 | Describe sources of financial or non-financial support for the review, and the role of the funders or sponsors in the review. | P29 Acknowledgements |
| Competing interests | 26 | Declare any competing interests of review authors. | P29 Conflicts of Interest |
| Availability of data, code and other materials | 27 | Report which of the following are publicly available and where they can be found: template data collection forms; data extracted from included studies; data used for all analyses; analytic code; any other materials used in the review. | P32 Appendices |

*From:*  Page MJ, McKenzie JE, Bossuyt PM, Boutron I, Hoffmann TC, Mulrow CD, et al. The PRISMA 2020 statement: an updated guideline for reporting systematic reviews. BMJ 2021;372:n71. doi: 10.1136/bmj.n71
